# Treatment Summary and Care Plan for Breast Cancer Survivors: acceptability and feasibility study*

**DOI:** 10.1590/1518-8345.7476.4620

**Published:** 2025-07-28

**Authors:** Maria das Graças Silva Matsubara, Fabiana Baroni Alves Makdissi, Simone Elias, Cristiane Decat Bergerot, Kimlin Tan Ashing, Edvane Birelo Lopes De Domenico

**Affiliations:** 1 AC Camargo Cancer Center, São Paulo, SP, Brazil.; 2 Univerdade Federal de São Paulo, São Paulo, SP, Brazil.; 3 Oncoclínicas, Brasília, DF, Brazil.; 4 City of Hope National Medical Center, California, Duarte, CA, United States of America.

**Keywords:** Breast Neoplasms, Cancer Survivors, Feasibility Studies, Patient Care Planning, Sickness Impact Profile, Medical Oncology

## Abstract

to evaluate the acceptability and feasibility of the Treatment Summary and Care Plan for Breast Cancer Survivors (TSSCP-P Br) document.

this was a cross-sectional, quantitative and qualitative study involving women who had completed treatment for breast cancer (n=50) and nurses (n=10) who incorporated the document into the care plan at outpatient follow-up appointments during the experimental phase of a clinical study. The feasibility and acceptability questionnaires were administered in the last data collection stage of the experiment. The data was analyzed using descriptive statistics and content analysis.

in the evaluation of the surviving women, feasibility showed retention of 98.0% and adherence of 99.3%. Acceptability in terms of suitability, convenience, efficacy and adherence reached 81.6%. From the professionals’ perspective, feasibility and acceptability were 84.2%, in terms of suitability, convenience, effectiveness, risks, availability, training, fidelity, reach and resources. The document was praised and the main problems in practice were pointed out, such as professional experience.

the Treatment Summary and Care Plan proved to be feasible and acceptable for the clinical practice of caring for women who have survived breast cancer.

## Highlights

**(1)** The Care Plan contributes to the cancer survivor’s self-management. **(2)** The Care Plan promotes person-centered care for cancer survivors. **(3)** Cancer survivors evaluated the Care Plan as a post-treatment right. **(4)** Nurses considered the Care Plan to be adequate, convenient and effective. **(5)** The implementation of the Care Plan requires specific oncology skills.

## Introduction

Breast cancer is the most common cancer among women worldwide, with more than 2 million annual diagnoses and, in Brazil, with 73,000 new cases estimated for the three-year period 2023-2025^(1-2)^.

These numbers are expected to increase due to the ageing of the population and the growing trends in modifiable risk factors for breast cancer, which will consequently result in an increase in the population of survivors, especially as a result of early diagnosis and effective combined treatments^(1)^. Currently, global 5-year survival rates range from over 90% in developed countries to 40-66% in middle- and low-income countries, respectively^(3)^.

This epidemiological scenario has resulted in new challenges to provide the best possible care for the growing population of survivors^(4)^. Conceptually, a cancer survivor is a person who has had cancer and is on the journey from diagnosis to treatment or beyond, throughout life^(5)^.

As a long-term survivor, there is a false perception that, at the end of treatment, the survivor will show an overall improvement in signs and symptoms over time. However, breast cancer survivors can experience late and long-term complications due to the disease and the treatments, surgery, antineoplastic chemotherapy and/or radiotherapy. These adverse effects can result in problems related to mental, physical and social health, culminating in compromised quality of life (QoL)^(6)^.

Therefore, in order to improve QoL, interventions appropriate to health needs should be provided, including guidance on managing long-term side effects, signs of recurrence, mental health, follow-up appointments and a healthy lifestyle^(7)^. However, many survivors lack complete and reliable information about becoming ill with cancer, given the need to understand its late manifestations and to report the journey so that professionals are able to treat adverse effects^(8)^.

The National Coalition for Cancer Survivorship (NCCS), a non-profit organization led by American cancer survivors that aims to advocate for quality care for people with cancer, states that all cancer survivors should have a treatment summary and care plan that addresses post-treatment needs to improve health and QoL once the initial cancer treatment has ended^(5)^. Several studies have already been carried out on the care plan for cancer survivors and have indicated the potential of the resource after the initial treatment has ended^(7,9-10)^.

Therefore, a national version was obtained from the translation and cross-cultural adaptation of the Treatment Summary and Survivorship Care Plan (TSSCP-S) for Brazilian breast cancer survivors, resulting in the Treatment Summary and Care Plan for Breast Cancer Survivors (TSSCP-P Br), consisting of: what a survivorship care plan is, its importance, how to use it, information about breast cancer, data on cancer diagnosis and treatment, follow-up care and surveillance, care team, health advice and questions about QoL^(11)^.

The TSSCP-P Br has had a positive impact on the self-efficacy, physical and emotional well-being of breast cancer survivors^(12)^. In this Brazilian study, the aim was to assess the acceptability and feasibility from the perspective of both the professionals and the recipients of the intervention at the end of the intervention, in order to estimate the potential for its application in the real world, as well as enabling adjustments in future studies and practices; these are the data presented in the present investigation.

It is now increasingly recognized that acceptability and feasibility should be considered when designing, evaluating and implementing health interventions^(13-14)^. Feasibility confirms whether the intervention can be implemented as planned, while acceptability indicates whether potential recipients are willing and able to receive and adhere to the intervention^(13,15)^.

There is a global trend to search for evidence on the ability to implement care plans for cancer survivors^(9)^, which justifies the intentionality of the present study. Thus, acceptability in this investigation referred to the way in which patients and health professionals received and used the TSSCP-P Br, while feasibility related to the potential for implementing the plan in practice and its ability to support continuity of care. The objectives were to assess the acceptability and feasibility of the Treatment Summary and Care Plan for Breast Cancer Survivors (TSSCP-P Br) document.

## Method

### Study design

This is a cross-sectional, quantitative and qualitative study, part of an extensive research project^(11-12)^. This investigation used guidelines that are widely recommended for studies that aim to assess acceptability and feasibility^(13,15-16)^ and in accordance with the Revised Standards for Quality Improvement Reporting Excellence (SQUIRE 2.0) tool.

### Setting and period

The study was carried out at a Cancer Center in the city of São Paulo, SP, Brazil, from June 2021 to May 2022. The host institution is philanthropic, with care linked to the Unified Health System, with free and universal access, and Supplementary Health, linked to companies providing health services.

### Population and criteria for selecting and defining participants

The participants in this study were 50 breast cancer survivors of the 51 who consented to take part and received the TSSCP-P Br, as well as 10 nurses who provided guidance on its purpose and use. The inclusion criteria for selecting the patients were: belonging to the experiment group, aged over 18, female, diagnosed with breast cancer, at any pathological stage, undergoing clinical therapies with antineoplastic chemotherapy, radiotherapy and surgical treatment, treated exclusively at the study’s host institution, who had finished their treatment (except endocrine therapy). Patients were excluded if they had not undergone surgical procedures to treat breast cancer; if they had a history of other cancers, except non-melanoma skin cancer; if they were not fluent in Portuguese and if they had psychiatric disorders attested to in their medical records.

The nurses who took part in the intervention were recruited from the Multiprofessional Residency Program and Permanent Education at the study’s host institution. To be eligible, they had to have a postgraduate degree in oncology and/or be studying specialization in the residency modality; declare their availability and take part in the training to apply the TSSCP-P Br.

With regard to sample calculation, the specialized literature shows that a minimum of 30 participants is considered appropriate for studies aimed at assessing whether an intervention is appropriate^(16-17)^.

### Study variables

The dependent variables were the acceptability and feasibility of the TSSCP-P Br, while the independent variables included sociodemographic data (age, marital status, level of education, religious belief, socioeconomic classification and experience in oncology) and clinical data (histological type of breast cancer, staging and treatment).

### Instruments used to collect information

The instruments for assessing acceptability by patients and professionals were drawn up based on the precepts of complex health interventions by Sidani and Braden^(15)^, as well as the theoretical framework of acceptability by Sekhon, et al.^(14)^, opting for common criteria and those closely related to the objectives of this investigation.

The instrument for assessing feasibility by professionals was based on the Structured Assessment of Feasibility (SAFE), a standardized measure for assessing the feasibility of implementing complex interventions in the mental health services of the National Health Service (NHS), but which can be applied in a variety of studies, from simple pharmacological interventions to complex institutional innovations, without the need to obtain authorization, as long as it is duly cited, as instructed by the authors^(18)^ (questionnaires available at: https://bit.ly/3wXe0UU).

The assessment of feasibility by patients was based on adherence and retention rates^(14)^.

The questionnaire designed to assess patient acceptability consisted of four dimensions and 11 items: (1) Suitability (three items); (2) Convenience (two items); (3) Effectiveness (four items); (4) Adherence (two items).

At the end of the questionnaire there was space for comments, suggestions and the following questions: What did you find most interesting and what do you consider to be a positive point? What would you change?

Since the respondents wrote the questionnaire in their own handwriting, validation took place on the day of data collection, in an attempt to clarify eligible content and content that was difficult to interpret, thus legitimizing the answers with the respondents.

The questionnaire for assessing acceptability and feasibility by nurses was made up of ten dimensions and 22 items: (1) Suitability (three items); (2) Convenience (two items); (3) Effectiveness (one item); (4) Risks (two items); (5) Adherence (two items); (6) Availability, quantity and skill of human resources (three items); (7) Training (one item); (8) Material, technological and physical resources (three items); (9) Loyalty (four items) and; (10) Reach (one item). At the end of the questionnaire there was space for comments and suggestions. The content of the comments and suggestions was validated afterwards by e-mail.

The dimensions of both questionnaires were assessed using a 5-point Likert scale: 1) Strongly disagree; 2) Disagree; 3) Neutral; 4) Agree; 5) Strongly agree.

### Data collection

The TSSCP-P Br was applied in two individual meetings, three months apart and lasting between 30 and 90 minutes. At the first meeting, the participants received the TSSCP-P Br in booklet format and watched an explanatory video covering the content of the care plan. The participants also provided demographic information, which was not included in the PEP. In the second meeting, doubts were clarified and the skills acquired were reinforced, as well as the development of new skills for dealing with late effects, lasting between 20 and 60 minutes^(12)^.

At the third and final meeting, after six months of testing the TSSCP-P Br, the acceptability questionnaire was administered to women who had survived breast cancer and had taken part in the two previous meetings.

The nurses took part in a two-hour theoretical-practical face-to-face training session, conducted by the main researcher, in which the TSSCP-P Br was explained, and a script and video containing the approach to be taken with the patient were discussed and made available. The learning objectives of the training were to learn about the epidemiological data, risk factors, etiology and diagnosis of breast cancer; to understand the classification, staging and treatment of breast cancer; to understand about cancer survivorship and the importance of the care plan; to learn about the TSSCP-P Br; to apply care based on the TSSCP-P Br and to complete the TSSCP-P Br.

The professionals were emailed a link to access the feasibility questionnaire available on the Google Forms[^®^]{dir=“rtl”} platform immediately after the end of the TSSCP-P Br application period.

### Data analysis

Descriptive statistics were used for the quantitative variables, in absolute and relative numbers. It was determined a priori that the intervention would be considered viable and acceptable if the indicators achieved a result of > 80%^(16)^.

When assessing feasibility for women survivors, the retention rate (number of participants present during the three data collection times of the experimental study x 100/number of participants who agreed to take part) and adherence rate (number of times completed x 100/total number of times) were taken into account^(14)^. To assess feasibility from the nurses’ perspective and acceptability to women survivors, the favorable options on the Likert scale were considered (4 and 5 when agreement was expected and 1 and 2 when disagreement was expected).

Bardin’s content analysis was used for the qualitative data related to the perception of breast cancer survivors and professionals about the TSSCP-P Br, following the structured steps consisting of: pre-analysis, exploration of the material and treatment of the results, inference and interpretation^(19)^. The data generated was selected, analyzed and categorized using ATLAS.ti 8.0 software (Scientific Software Development, Berlin, Germany). Data interpretation was based on the conceptual and operational bases of Sidani and Braden’s complex interventions^(15)^.

### Ethical aspects

The study was approved by the Research Ethics Committees of the Federal University of São Paulo and the Antônio Prudente Foundation, protocol numbers 3.203.556/2019 and 3.351.638/2019. All participants provided informed consent.

## Results

Fifty women took part in the study, all linked to the Supplementary Health System, and 10 nurses. For a better understanding of the findings, the data from the acceptability and feasibility study from the perspective of the women survivors is presented first, followed by the feasibility study from the point of view of the professionals.

The sociodemographic data shows that the average age of the participants was 55.7 years (standard deviation 12.1 years), ranging from 36 to 81 years. The majority (66.0%) were married or living with a partner, 74.0% had higher education and 60.0% identified themselves as Catholic. In terms of socio-economic classification, 60.0% belonged to class A and 32.0% to class B. In terms of clinical data, most of the participants had non-special invasive carcinoma (72.0%), with pathological stages I (34.0%) and II (32.0%). The most common treatments included a combination of surgery and radiotherapy (54.0%) and surgery, antineoplastic chemotherapy and radiotherapy (32.0%).

The feasibility analysis revealed a retention rate of 98.0%, covering 50 of the 51 participants who consented to take part in the study; and adherence reached 99.3% in the three data collection times relating to the experimental study.

The overall acceptability rate was 81.6%, with 93.3% for suitability, 85.0% for convenience, 73.5% for effectiveness and 74.0% for adherence (Table 1).

Of the participants, 39 (78.0%) answered one of the two questions which, to recall, consisted of asking about interesting, positive and modifiable topics, and whether they wanted to write comments.

The data was read and organized according to the nature of the content by semantic similarity, inferential interpretation was carried out and the frequency of the recording units was counted. The contents of the quotes corresponding to the inferential analyses and their respective codes regarding women’s perceptions of the TSSCP-P Br are shown in Figure 1.

**Table 1 -:** Acceptability of the TSSCP-P Br* by women breast cancer survivors (n^†^= 50). São Paulo, SP, Brazil, 2022

**Dimension**	**Affirmative**	**Strongly disagree**	**Disagree**	**Neutral**	**Agree**	**Totally agree**
		**n** ^†^ **(%)**	**n** ^†^ **(%)**	**n** ^†^ **(%)**	**n** ^†^ **(%)**	**n** ^†^ **(%)**
Suitability	The guidance provided by the professional was adequate.	0 (0.0)	0 (0.0)	2 (4.0)	11 (22.0)	37 (74.0)
	The information contained in the care plan is clear and easy to understand.	0 (0.0)	0 (0.0)	3 (6.0)	16 (32.0)	31 (62.0)
	The size and type of font, as well as the presentation in printed format and spiral model, are appropriate.	0 (0.0)	0 (0.0)	2 (4.0)	11 (22.0)	37 (74.0)
Convenience	The recommendations and guidelines are easy to follow.	0 (0.0)	0 (0.0)	5 (10.0)	11 (22.0)	34 (68.0)
	The plan contains information that is new to you.	2 (4.0)	1 (2.0)	7 (14.0)	18 (36.0)	22 (44.0)
Effectiveness	The information contained in the plan can bring benefits to your health and well-being.	0 (0.0)	0 (0.0)	2 (4.0)	15 (30.0)	33 (66.0)
	The information contained in the care plan provided some decision-making to plan appropriate follow-up care for you.	0 (0.0)	0 (0.0)	6 (12.0)	21 (42.0)	23 (46.0)
	You feel confident in following the recommendations contained in the care plan.	0 (0.0)	0 (0.0)	2 (4.0)	13 (26.0)	35 (70.0)
	You felt scared or uncomfortable when reading the care plan.	22 (44.0)	12 (24.0)	9 (18.0)	4 (8.0)	3 (6.0)
Adherence	You will adopt the care plan as a short- and long-term guide to follow up on your health and well-being.	1 (2.0)	0 (0.0)	4 (8.0)	17 (34.0)	28 (56.0)
	Some session/part of the care plan does not seem useful to you.	16 (32.0)	13 (26.0)	9 (18.0)	8 (16.0)	4 (8.0)

*TSSCP-P Br = Summary of Treatment and Care Plan for Breast Cancer Survivor; ^†^ n = Number of participants

The evaluation of the feasibility of the TSSCP-P Br from the professional’s perspective involved ten nurses, with a mean age of 30.6 years (minimum 23 and maximum 50), experience in oncology with a mean of 5.6 years, ranging from one to 21 years, 60.0% with postgraduate degrees and 40.0% studying for a residency in oncology.

Overall feasibility was 84.2%, with 100.0% for suitability; 95.0% for convenience; 100.0% for effectiveness; 56.7% for risks; 70% for adherence; 70% for availability, quantity and ability of human resources; 90% for material, technological and physical resources; 97.5% for fidelity and 90% for reach (Table 2).

**Figure 1 -:** Contents of quotes corresponding to inferential analyses and their respective codes regarding women’s perception of the TSSCP-P Br*. São Paulo, SP, Brazil, 2022

**ID**	**Citation content**	**Inferential analysis**	**Categories**
17:2; 19:4; 20:2; 24:1; 25:2; 26:2; 30:4; 31:2; 38:2; 39:3; 40:3; 42:1; 45:2; 47:3; 49:2; 57:1	*Nothing to add.*	Absolute validation of presentation and content	Acceptability and viability ensured
55:1	*I wouldn’t change anything, it’s very well prepared.*		
48:3	*It’s very well prepared.*		
34:1; 36:3; 48:2	*I wouldn’t change anything.*		
22:2	*I used it as a guide for my treatment (a summary of the whole process during medical appointments).*	Guidance and direction	Viability: guides and encourages self-management
46:2	*It’s very useful post-treatment for adapting to the new normal. There are many specialties and deadlines that we can’t miss.*		
37:1	*[...] to help and guide patients during treatment.*		
42:2	*Application and guidelines.*	Guidance and direction	Viability: guides and encourages self-management
21:1	*I was able to clarify my care and find out what was happening to me.*		
47:2	*[...] tips that are given post-surgery are also very important.*	Longitudinal and complete	Acceptability: summarizes important information
47:2	*[...] tips that are given post-surgery are also very important.*		
49:1	*[...] I’m interested in improving care and monitoring not only of the disease itself, but also of our psychological state.*		
16:3	*[...] all the important information about the treatment in one place.*		
30:2	*A lot of clarification.*		
35:1	*[...] information is extremely important.*		
40:1	*[...] important information for treatment.*		
30:2	*A lot of information.*		
26:1	*[...] information about my treatment (positive point)*		
28:1	*[...] having the complete history of my diagnosis (positive point).*		
47:1	*I found the account of my entire history since the day of my surgery interesting.*		
29:2	*I found the material to be good quality, informative and practical.*		
36:2	*[...] care and applicability in everyday life.*		
39:1	*[...] making it clear that it is possible to have a life after cancer, to feel like a woman.*	Positive feelings: knowledge, security, self-confidence, recognition of initiative, of the need to be a right	Acceptability: valuable
39:2	*[...] the plan has done me good.*		
39:4	*Keep doing this work. After receiving the plan, I did a photo shoot.*		
58:1	*The guidance on how to get on with life with confidence and joy is very valuable.*		
45:1	*[...] the content is very helpful.*		
47:4	*Congratulations on your excellent work and your care.*		
45:3	*I thought the idea was very good, I suggest you continue.*		
56:1	*[...] Cancer survivors are very strong.*		
42:3	*Everyone with cancer should receive the manual.*		
39:1	*[...] make it clear that it is possible to have a life after cancer, to feel like a woman.*		
32:2	*[...] I liked it.*		
33:2	*[...] security.*	Positive feeling: security	
18:2	*Many things to learn.*	Enables learning	Acceptability: educational
23:1	*I liked the organizations and the explanations.*		
53:2	*It made me learn.*		
25:1	*[...] clarification of management and post-treatment care.*		
54:1	*[...] improved knowledge about the disease.*		
36:1	*Easy-to-understand language.*	Appropriate content and language	Feasibility: didactic
22:1	*Organization of the material and the way in which it provides guidance.*		
38:1	*Easy to read, objective texts.*		
40:2	*[...] clear information.*		
46:1	*The layout of the subjects is easy to use.*		
21:2	*Clear language of terms (positive point).*		
46:1	*The layout of the subjects is easy to use.*		
17:1	*[...] clarifications are objective.*	Beyond the biological	Acceptability: holistic
41:1	*[...] I can’t remember everything I read.*		
43:1	*Positive point: health and well-being.*		
20:1	*[...] care of the mind.*		
41:2	*[...] great for those starting treatment.*	Best applied at the start of treatment	Acceptability and feasibility relativized: anticipate delivery
41:3	*[...] superficial for those in the tamoxifen stage.*	Superficial in some respects	Acceptability and feasibility relativized: incomplete
41:4	*[...] pandemic and flu crisis and this health control could also be included in the manual.*		
54:2	*There should be a booklet suitable for each case.*	Personalization/ Individualization	Acceptability and feasibility relativized: individualize
28:2	*I wouldn’t use the word survivor.*	Uncomfortable with the term “survivor”	Acceptability and viability relativized: survivor as denomination
56:1	*Cancer survivor is too strong.*		
48:1	*I didn’t read the plan because I don’t like reading and I don’t like remembering my illness.*	Excessive amount of content	Acceptability and feasibility relativized: extension

*TSSCP-P Br = Summary of the Treatment and Care Plan for Breast Cancer Survivors

**Table 2 –:** Evaluation of the acceptability and feasibility of the TSSCP Br*, from the perspective of professionals (n^†^= 10). São Paulo, SP, Brazil, 2022

**Dimension**	**Affirmative**	**Evaluation**				
		**Strongly disagree**	**Disagree**	**Neutral**	**Agree**	**Totally agree**
		**n** ^†^ **(%)**	**n** ^†^ **(%)**	**n** ^†^ **(%)**	**n** ^†^ **(%)**	**n** ^†^ **(%)**
Suitability	The objectives of the intervention correspond to those prioritized for women who are breast cancer survivors.	0 (0.0)	0 (0.0)	0 (0.0)	3 (30.0)	7 (70.0)
	The recommendations and guidelines are applicable to the population of breast cancer survivors.	0 (0.0)	0 (0.0)	0 (0.0)	3 (30.0)	7 (70.0)
	The way in which the intervention is being implemented can generate the desired objectives.	0 (0.0)	0 (0.0)	0 (0.0)	3 (30.0)	7 (70.0)
Convenience	The intervention is easy to implement in everyday practice.	0 (0.0)	1 (10.0)	0 (0.0)	4 (40.0)	5 (50.0)
	The team requires specific training to carry out the intervention.	0 (0.0)	0 (0.0)	0 (0.0)	4 (40.0)	6 (60.0)
Effectiveness	The intervention is effective, both in the short and long term.	0 (0.0)	0 (0.0)	0 (0.0)	4 (40.0)	6 (60.0)
Risks	The intervention may cause harm or discomfort to the breast cancer survivor.	5 (50.0)	2 (20.0)	2 (20.0)	1 (10.0)	0 (0.0)
	The intervention causes risk for the health professional.	9 (90.0)	1 (10.0)	0 (0.0)	0 (0.0)	0 (0.0)
Adherence	The breast cancer survivor will use the material to improve surveillance and health-related care.	0 (0.0)	0 (0.0)	1 (10.0)	4 (40.0)	5 (50.0)
	The health team will adopt the intervention into their daily practice.	0 (0.0)	0 (0.0)	3 (30.0)	5 (50.0)	2 (20.0)
Availability, quality and skill of human resources	The intervention requires additional human resources.	0 (0.0)	2 (20.0)	3 (30.0)	3 (30.0)	2 (20.0)
	There are professionals available to implement the intervention.	0 (0.0)	1 (10.0)	1 (10.0)	7 (70.0)	1 (10.0)
	The intervention requires specialized human resources.	1 (10.0)	0 (0.0)	1 (10.0)	5 (50.0)	3 (30.0)
Training	The multi-professional team is trained to implement the intervention.	0 (0.0)	2 (20.0)	0 (0.0)	4 (40.0)	4 (40.0)
Material, technological and physical resources	The care plan in printed form is adequate.	0 (0.0)	1 (10.0)	0 (0.0)	1 (10.0)	8 (80.0)
	Technological resources for accessing information about the intervention are adequate.	0 (0.0)	0 (0.0)	0 (0.0)	3 (30.0)	7 (70.0)
	The physical environment for the intervention is adequate.	0 (0.0)	1 (10.0)	1 (10.0)	4 (40.0)	4 (40.0)
Fidelity	The content of the care plan is easy to understand.	0 (0.0)	0 (0.0)	0 (0.0)	4 (40.0)	6 (60.0)
	The activities related to filling in the care plan are easy.	0 (0.0)	0 (0.0)	1 (10.0)	5 (50.0)	4 (40.0)
	The activities related to the applicability of the plan are easy.	0 (0.0)	0 (0.0)	0 (0.0)	4 (40.0)	6 (60.0)
	The time taken to carry out the intervention, considering completion and application, is adequate.	0 (0.0)	0 (0.0)	0 (0.0)	7 (70.0)	3 (30.0)
Reach	The activities cover care in the various stages of survival.	0 (0.0)	0 (0.0)	1 (10.0)	4 (40.0)	5 (50.0)

*TSSCP-P Br = Summary of Treatment and Care Plan for Breast Cancer Survivor; ^†^n = Number of participants

Of the ten participants, seven (70%) wrote comments on the TSSCP-P Br. The content analysis technique was used in the same way as for the group of female survivors. The contents of the quotes corresponding to the inferential analyses and their respective codes, regarding the professionals’ perception of the TSSCP-P Br are shown in Figure 2.

**Figure 2 -:** Contents of quotes corresponding to inferential analyses and their respective codes, referring to professionals’ perception of the TSSCP-P Br*. São Paulo, SP, Brazil, 2022

**ID**	**Citation content**	**Inferential analysis**	**Categories**
4:5	*[...] information in the palm of your hand.*	Job satisfaction	Acceptability: contributes to professional practice
4:3	*[...] bag size.*		
4:6	*[...] digital or physical version.*		
1:1	*It was an incredible experience to be able to contribute and learn a lot from everyone*		
3:4	*[...] expressions of gratitude and welcome from the family and the patient themselves.*		
3:7	*The opportunity to be part of this process.*		
4:1	*[...] enjoyable.*		
7:1	*A very special personal and professional experience.*	Job satisfaction	Acceptability: contributes to professional practice
2:5	*[...] a “link” between patient and professional.*		
3:3	*[...] the plan encourages questions.*	Encourages work activity	Acceptability: contributes to professional practice
5:3	*Monitoring the progress of treatment.*		
5:2	*[...] facilitator for future consultations.*		
2:1	*[...] a great tool, not just for information [...]*		
3:5	*[...] in the patients I approached(...) there were expressions of gratitude and welcome, involving family and patient.*	Quality of care	Feasibility: patient- and family-centered care
2:4	*[...] it brings warmth and care to the patient.*		
6:1	*[...] the health professional’s view of the survivor who has already gone through the most difficult process of the disease: discovery, treatment and physical and social suffering.*		
6:2	*[...] empathetic. We professionals forget that the survivor’s life changes. It’s necessary to give them guidance on how to lead their lives differently.*		
5:1	*Fundamentally important for the patient, as it provides information from the start of treatment.*		
3:5	*[...] in the patients I approached [...] there were expressions of gratitude and welcome, involving family and patient.*		Viability: considers the implications of the cancer disease on the survivor
4:4	*[...] form of app.*	Using digital technology	Relativized acceptability: form of presentation
4:5	*Information in the palm of your hand.*	Compressed presentation	
4:3	*[...] bag size.*		
4:6	*Digital or physical version.*	With more than one presentation	
4:2	*The Plan contains all the information, however, it could be more compact.*	Complete, with the potential to be summarized	Relativized viability: too much content
3:1	*[...] two patients felt uncomfortable with the word survivor*	The word survivor can create discomfort in the patient	Relativized viability: survivor as denomination

*TSSCP-P Br = Summary of Treatment and Care Plan for Breast Cancer Survivors

## Discussion

The study presented data that reinforces the acceptance and viability of the TSSCP-P Br as a viable resource and qualifier of care for women who have survived breast cancer. The majority reported that the plan contained new information, that the information it contained provided some decision-making for planning follow-up care and that the recommendations and guidelines were easy to carry out. In a study evaluating the impact of providing care plans for survivors, it was also found that the majority of participants considered the information in the plan to be new^(9,20)^.

Cancer survivors value interventions with content specific to their needs, that are easy to use, accessible and delivered at the right time during the cancer journey^(21)^. It is important to note that needs and preferences vary from person to person at all stages of the journey, including the post-treatment period, and that all survivors need comprehensive and relevant information to guide them in managing their health^(7,20)^.

As found in this study, the TSSCP-P Br can be adopted as a very useful guide in all its constituent parts, in the short and long term of survival, to follow up on health and well-being. The data corroborates studies which indicate that improving patients’ knowledge of their illness and treatment generates greater engagement with the health management process^(22-23)^. This was also in line with the satisfactory results in terms of feasibility, with high adherence and retention, which demonstrated greater participation in the health self-management process.

For the nurses involved in the implementation, the TSSCP-P Br was considered viable, although some limiting factors were pointed out, especially in relation to the number of professionals and their technical specificity in oncology. The need for additional human resources can be justified by the time needed to deliver the plan, which can vary from 20 to 90 minutes according to the literature^(20)^. In addition, the inadequate training of professionals to care for cancer survivors is also a widely discussed limiting factor which can have a negative impact on clinical practice^(20)^. It is important to note that the perception of professionals when applying an intervention is influenced by various factors, such as personal values and beliefs, professional training, theoretical knowledge, practical experience and the use of good practice guidelines^(16)^.

When professionals consider an intervention to be unacceptable to patients, they may avoid it. Therefore, the perception of the ability to carry it out, as well as the practicality of its application, can increase the motivation to carry out the intervention, impacting on the loyalty to adopt the actions contained in the plan^(17)^.

The implementation of successful interventions depends on their acceptability by users. Acceptability is a multifaceted construct that reflects the extent to which people who carry out or receive a health intervention consider it appropriate. The theoretical framework of acceptability is made up of seven components: affective attitude, burden, applicability, ethics, coherence of the intervention, costs and self-efficacy^(12)^.

The analytical possibilities of the statements showed that the discursive responses were extremely enlightening, as they revealed perceptions that could not be explored in the Likert scale responses, increasing the validity of the findings^(9,15)^.

In the qualitative analysis of statements about the attributes of the TSSCP-P Br, from the perspective of women who are breast cancer survivors, categories were generated that expressed various qualities of the tool. This population values access to self-care information organized on the basis of the cancer continuum, in order to support self-management^(24)^.

From the point of view of the professionals who applied the intervention, several positive aspects were also highlighted, mainly as a possibility of materializing patient-centred care. In a study aimed at developing and evaluating the TSSCP-S, the evaluators (professionals and breast cancer survivors) noted that its use promoted patient-centered care^(25)^.

Person-centered care is among the main models that contribute to excellence in care^(26)^, advocated by health regulatory institutions and those that have hospital accreditation programs, such as the Brazilian Accreditation System (SBA-ONA), Joint Commission International (JCI), based on the North American model, and Accreditation Canada International (ACI), recently renamed the Health Standards Organization (HSO)^(27)^.

It should be noted that the institution where the study was carried out is accredited by Accreditation Canada International and this means that the results of this research, when incorporated into everyday practice, will boost achievements to even higher levels.

In addition, for both respondents, negative aspects of the TSSCP-P Br were revealed, such as the possible suffering generated by information that anticipates facts and risks, the generality and extent and the use of the term “survivor”. The term “cancer survivor” is widely used by different people, health institutions, academic bodies and political organizations. However, in many countries, patients interpret these terms negatively, associating them with the memory of the high risk of death and the dissociation with a cure, which generates rejection, as they continue to deal with the fear of recurrence^(28)^.

Comparing the findings with the literature, a meta-analysis that examined the feasibility of implementing care plans for cancer survivors, from the perspectives of survivors and health professionals, concluded that the plans are acceptable and valued by both^(20)^. In short, assessing acceptability and feasibility can help to identify facilitators and barriers to implementing the intervention and understand the achievement of the expected results, as attested to in the vast literature on the subject^(16,29)^.

Among the limitations, we highlight the fact that the data was collected in a single location and that the institution in question is a cancer center with hospital accreditation. Unfortunately, this is not the reality of most public cancer treatment centers in Brazil, which are located in general hospitals, not accredited in Oncology, with a limited number of specialized professionals and poor physical structures for excellent care.

Another important limitation is the sociodemographic characterization of breast cancer survivors, who have a different profile from the national scenario, making it difficult to generalize the results to survivors exclusively from the Brazilian public health system. A larger sample of professionals should also be considered in a future study to confirm the findings.

From the perspective of the contributions of this study to the improvement of nursing science, it is worth highlighting the importance of carrying out studies aimed at assessing the acceptability and feasibility of innovative proposals. The acceptability and feasibility study has in fact added a set of data that could help refine the application of the TSSCP-P Br in the care of women who have survived breast cancer after the end of their initial treatment.

## Conclusion

The quantitative data from this study indicated that the TSSCP-P Br achieved satisfactory levels of acceptability and feasibility. From the point of view of breast cancer survivors, feasibility showed high values for retention and adherence, as well as for acceptability in all dimensions. From the professionals’ perspective, acceptability and feasibility also showed high values in almost all the dimensions evaluated.

In the qualitative analysis, women breast cancer survivors praised the TSSCP-P Br, showing how valuable it is, how educational it is, how it can summarize important information, encourage self-management and be didactic. However, there was also the risk of it being incomplete or long, and they expressed discomfort with the use of the term “survivor”.

The nurses’ assessment of TSSCP-P Br was also positive, as it encourages qualified professional practice, patient- and family-centered care, and can bring together the complexity of cancer in the context of survivorship. However, the professionals suggested the possibility of compacting content, making it available in digital format and warned against the risk of the term “survivor” generating discomfort in patients.
