# Supplementary material for: Treatment Summary and Care Plan for Breast Cancer Survivors: acceptability and feasibility study
Source: Rev Lat Am Enfermagem. 2025 Jul 28;33:e4620. doi: 10.1590/1518-8345.7476.4620 (PMC12306929; doi:10.1590/1518-8345.7476.4620)
Supplement: Translation

# Translation: Resumo do Tratamento e Plano de Cuidados para Sobreviventes de Câncer de Mama: estudo de aceitabilidade e viabilidade*

**DOI:** 10.1590/1518-8345.7476.4621

**Authors:** Maria das Graças Silva Matsubara, Fabiana Baroni Alves Makdissi, Simone Elias, Cristiane Decat Bergerot, Kimlin Tan Ashing, Edvane Birelo Lopes De Domenico

**Affiliations:** 1 AC Camargo Cancer Center, São Paulo, SP, Brasil.; 2 Univerdade Federal de São Paulo, São Paulo, SP, Brasil.; 3 Oncoclínicas, Brasília, DF, Brasil.; 4 City of Hope National Medical Center, California, Duarte, CA, Estados Unidos da América.

**Keywords:** Neoplasias da Mama, Sobreviventes de Câncer, Estudos de Viabilidade, Planejamento de Assistência ao Paciente, Perfil de Impacto da Doença, Oncologia

## Abstract

avaliar a aceitabilidade e a viabilidade do documento Resumo do Tratamento e Plano de Cuidados para a Sobrevivente de Câncer de Mama (TSSCP-P Br).

estudo transversal, quanti-qualitativo, com a participação de mulheres que finalizaram o tratamento para o câncer de mama (n=50) e enfermeiros (n=10) que incorporaram o documento ao plano de cuidados nas consultas de seguimento ambulatorial durante a fase experimental de um estudo clínico. Os questionários de viabilidade e aceitabilidade foram aplicados na última etapa de coleta de dados do experimento. Os dados foram analisados por meio da estatística descritiva e análise de conteúdo.

na avaliação das mulheres sobreviventes, a viabilidade demonstrou retenção de 98,0% e adesão de 99,3%. A aceitabilidade quanto à adequação, conveniência, eficácia e adesão atingiu 81,6%. Na perspectiva dos profissionais, a viabilidade e a aceitabilidade foram de 84,2%, quanto à adequação, conveniência, eficácia, riscos, disponibilidade, treinamento, fidelidade, alcance e recursos. O documento foi enaltecido e os principais problemas da prática foram apontados, como a experiência do profissional.

o Resumo do Tratamento e Plano de Cuidados mostrou-se viável e aceitável para a prática clínica do cuidado de mulheres sobreviventes do câncer de mama.

## Destaques

**(1)** O Plano de Cuidados contribui para o autogerenciamento do sobrevivente de câncer. **(2)** O Plano de Cuidados promove o cuidado centrado na pessoa sobrevivente de câncer.**(3)** Sobreviventes de câncer avaliaram o Plano de Cuidados como um direito pós-tratamento. **(4)** Enfermeiros consideraram o Plano de Cuidados como adequado, conveniente e eficaz. **(5)** A implementação do Plano de Cuidados exige competências específicas em oncologia.

## Introdução

O câncer de mama é o mais frequente entre as mulheres em todo o mundo, com mais de 2 milhões de diagnósticos anuais e, no Brasil, com 73 mil novos casos estimados para o triênio de 2023-2025^(1-2)^.

Prevê-se que esses números aumentem devido ao envelhecimento da população e às tendências crescentes dos fatores de risco modificáveis do câncer de mama, o que, consequentemente, resultará em um aumento da população de sobreviventes, especialmente em função dos diagnósticos precoces e tratamentos combinados eficazes^(1)^. Atualmente, as taxas globais de sobrevida em 5 anos variam em mais de 90% em países desenvolvidos e 40 a 66% em países de média e baixa rendas, respectivamente^(3)^.

Este cenário epidemiológico tem resultado em novos desafios para prestar o melhor atendimento possível à crescente população de sobreviventes^(4)^. Conceitualmente, o sobrevivente de câncer é a pessoa que teve câncer e que está na jornada do diagnóstico ao tratamento ou para além, ao longo da vida^(5)^.

Na condição de sobrevivente a longo prazo, existe a falsa percepção de que, ao término do tratamento, o sobrevivente apresentará melhora global dos sinais e sintomas com o passar do tempo. No entanto, sobreviventes de câncer de mama podem experienciar complicações tardias e a longo prazo devido à doença e aos tratamentos, cirurgia, quimioterapia antineoplásica e/ou radioterapia. Esses efeitos adversos podem resultar em problemas relacionados à saúde mental, física e social, que culminam em comprometimento da qualidade de vida (QV)^(6)^.

Assim, para melhorar a QV deve-se fornecer intervenções adequadas às necessidades de saúde, incluindo orientações sobre gestão dos efeitos colaterais a longo prazo, sinais de recorrência, saúde mental, consultas de acompanhamento e estilo de vida saudável^(7)^. Porém, muitos sobreviventes encontram-se desprovidos de informações completas e seguras sobre o adoecimento pelo câncer ante a necessidade de compreender as manifestações tardias que apresenta e para reportar a jornada para que os profissionais sejam capazes de tratar os efeitos adversos^(8)^.

A *National Coalition for Cancer Survivorship* (NCCS) – uma organização sem fins lucrativos liderada por sobreviventes de câncer da América, que visa defender cuidados de qualidade para as pessoas com câncer – afirma que todos os sobreviventes de câncer devem ter um resumo de tratamento e plano de cuidados que atenda às necessidades pós-tratamento para melhorar a saúde e QV, assim que findado o tratamento inicial do câncer^(5)^. Diversos estudos já foram realizados a partir do plano de cuidados para sobreviventes de câncer e indicaram as potencialidades do recurso após o término do tratamento inicial^(7,9-10)^.

Sendo assim, uma versão nacional foi obtida a partir da tradução e adaptação transcultural do *Treatment Summary and Survivorship Care Plan* (TSSCP-S) para sobreviventes brasileiras de câncer de mama, resultando no Resumo do Tratamento e Plano de Cuidados para a Sobrevivente de Câncer de Mama (TSSCP-P Br), composto por: o que é um plano de cuidados para a sobrevivência, sua importância, como utilizá-lo, informações sobre câncer de mama, dados sobre o diagnóstico e tratamento do câncer, cuidados de seguimento e vigilância, equipe de cuidados, conselhos de saúde e questões sobre QV^(11)^.

O TSSCP-P Br demonstrou impacto positivo na autoeficácia, bem-estar físico e emocional de sobreviventes de câncer de mama^(12)^. Nesse estudo brasileiro buscou-se, ao término da intervenção, avaliar a aceitabilidade e viabilidade na perspectiva tanto dos profissionais quanto dos receptores da intervenção, para estimar o potencial de sua aplicação no mundo real, além de possibilitar ajustes em estudos e práticas futuras; sendo estes os dados apresentados na presente investigação.

Na atualidade, é cada vez mais reconhecido que a aceitabilidade e a viabilidade devem ser consideradas ao se conceber, avaliar e implementar intervenções de saúde^(13-14)^. A viabilidade confirma se a intervenção pode ser implementada conforme planejado, enquanto a aceitabilidade indica se os destinatários potenciais estão dispostos e são capazes de receber e aderir à intervenção^(13,15)^.

Há uma tendência global de busca por evidências sobre a capacidade de implementação de planos de cuidados para sobreviventes de câncer^(9)^ que justifica a intencionalidade do presente estudo. Assim, a aceitabilidade, na presente investigação, referiu-se à forma como as pacientes e profissionais de saúde receberam e utilizaram o TSSCP-P Br, enquanto a viabilidade relacionou-se às potencialidades de implementação do plano na prática e sua capacidade de apoiar a continuidade do cuidado. Os objetivos foram avaliar a aceitabilidade e viabilidade do documento Resumo do Tratamento e Plano de Cuidados para a Sobrevivente de Câncer de Mama (TSSCP-P Br).

## Método

### Delineamento do estudo

Estudo transversal, quanti-qualitativo, parte integrante de um extensa pesquisa^(11-12)^. A presente investigação utilizou diretrizes amplamente recomendadas para estudos que intencionam avaliar aceitabilidade e viabilidade^(13,15-16)^ e de acordo com a ferramenta *Revised Standards for Quality Improvement Reporting Excellence* (SQUIRE 2.0).

### Local e período

O estudo foi realizado em um *Cancer Center* da cidade de São Paulo - SP, Brasil, no período de junho de 2021 a maio de 2022. A instituição-sede é filantrópica, com assistência vinculada ao Sistema Único de Saúde, de acesso gratuito e universal, e de Saúde Suplementar, vinculada às empresas prestadoras de serviços de saúde.

### População e critérios de seleção e definição dos participantes

As participantes deste estudo foram 50 mulheres sobreviventes de câncer de mama, das 51 que consentiram em participar e receberam o TSSCP-P Br, além de dez enfermeiras que orientaram sobre sua finalidade e uso. Os critérios de inclusão para a seleção das pacientes foram: pertencer ao grupo experimento, idade acima de 18 anos, sexo feminino, com o diagnóstico de câncer de mama, em qualquer estadiamento patológico, submetidas às terapias clínicas com quimioterapia antineoplásica, radioterapia e tratamento cirúrgico, atendidas exclusivamente na instituição-sede do estudo, que finalizaram o tratamento (exceto terapia endócrina). Foram excluídas as pacientes que não foram submetidas a procedimentos cirúrgicos para o tratamento do câncer de mama; com histórico de outros cânceres, exceto câncer de pele não melanoma; não ser fluente na língua portuguesa e portadoras de distúrbios psiquiátricos atestados em prontuário médico.

Em relação aos enfermeiros que participaram da intervenção, estes foram recrutados do Programa de Residência Multiprofissional e na Educação Permanente da instituição-sede do estudo. Para serem elegíveis, deveriam possuir pós-graduação em oncologia e/ou estar cursando a especialização na modalidade residência; declarar disponibilidade e participar do treinamento para a aplicação do TSSCP-P Br.

Em relação ao cálculo amostral, em literatura especializada evidencia-se que o adequado é o mínimo de 30 participantes para estudos que visam avaliar se uma intervenção é apropriada^(16-17)^.

### Variáveis do estudo

As variáveis dependentes foram a aceitabilidade e a viabilidade do TSSCP-P Br, enquanto as variáveis independentes incluíram os dados sociodemográficos (idade, estado civil, grau de instrução, crença religiosa, classificação socioeconômica e experiência em oncologia) e clínicos (tipo histológico do câncer de mama, estadiamento e tratamento).

### Instrumentos utilizados para a coleta das informações

Os instrumentos para avaliar a aceitabilidade pelas pacientes e pelos profissionais foram elaborados com base nos preceitos das intervenções complexas em saúde de Sidani e Braden^(15)^, bem como no referencial teórico de aceitabilidade de Sekhon, et al.^(14)^, optando-se pelos critérios comuns e por aqueles com estreita relação com os objetivos da presente investigação.

O instrumento destinado à avaliação da viabilidade pelos profissionais foi fundamentado no *Structured Assessment of Feasibility* (SAFE), uma medida padronizada para avaliar a viabilidade de implementação de intervenções complexas nos serviços de saúde mental do *National Health Service* (NHS), mas que pode ser aplicada em uma variedade de estudos, desde intervenções farmacológicas simples até inovações complexas institucionais, sem necessidade de obtenção de autorização, desde que devidamente citado, conforme orientado pelos autores^(18)^ (questionários disponíveis em: https://bit.ly/3wXe0UU).

A avaliação da viabilidade pelas pacientes foi conduzida com base nas taxas de adesão e retenção^(14)^.

O questionário destinado à avaliação da aceitabilidade pelas pacientes foi composto por quatro dimensões e 11 itens: (1) Adequação (três itens); (2) Conveniência (dois itens); (3) Eficácia (quatro itens); (4) Adesão (dois itens).

No final do questionário, havia espaço para comentários, sugestões e as seguintes perguntas: O que achou mais interessante e que você considera como ponto positivo? O que você modificaria?

Uma vez que as respondentes escreveram de próprio punho, a validação aconteceu no dia da coleta de dados, buscando esclarecer os conteúdos elegíveis e os de difícil interpretação, legitimando assim as respostas com as respondentes.

O questionário para as avaliações da aceitabilidade e viabilidade pelos enfermeiros foi composto por dez dimensões e 22 itens: (1) Adequação (três itens); (2) Conveniência (dois itens); (3) Eficácia (um item); (4) Riscos (dois itens); (5) Adesão (dois itens); (6) Disponibilidade, quantidade e habilidade dos recursos humanos (três itens); (7) Treinamento (um item); (8) Recursos materiais, tecnológicos e físicos (três itens); (9) Fidelidade (quatro itens); e (10) Alcance (um item). No final do questionário havia espaço para comentários e sugestões. A validação do conteúdo referente aos comentários e sugestões aconteceu *a posteriori* por meio de contato via e-mail.

As avaliações das dimensões de ambos os questionários foram realizadas por meio de uma escala tipo Likert de cinco pontos, sendo: 1. Discordo totalmente; 2. Discordo; 3. Neutro; 4. Concordo; 5. Concordo totalmente.

### Coleta de dados

A aplicação do TSSCP-P Br foi realizada por meio de dois encontros individuais, com intervalo de três meses entre eles e duração variando de 30 a 90 minutos. No primeiro encontro, as participantes receberam o TSSCP-P Br em formato de livreto e assistiram a um vídeo explicativo, abordando o conteúdo do plano de cuidados. As participantes também forneceram informações demográficas, que não constava do PEP. No segundo encontro, foram esclarecidas dúvidas e reforçadas as competências adquiridas, além do desenvolvimento de novas habilidades para lidar com os efeitos tardios, com duração de 20 a 60 minutos^(12)^.

No terceiro e último encontro, após o período de seis meses de testagem do TSSCP-P Br, foi aplicado o questionário de aceitabilidade às mulheres sobreviventes de câncer de mama que haviam participado dos dois encontros anteriores.

Os enfermeiros participaram de um treinamento teórico-prático, presencial, com carga horária de duas horas, realizado pela pesquisadora principal, no qual o TSSCP-P Br foi esclarecido, e um roteiro e um vídeo contendo a abordagem a ser realizada com a paciente foram discutidos e disponibilizados. Os objetivos de aprendizagem do treinamento foram conhecer os dados epidemiológicos, fatores de risco, etiologia e diagnóstico do câncer de mama; entender a classificação, estadiamento e tratamento do câncer de mama; compreender sobre sobrevivente de câncer e a importância do plano de cuidados; conhecer o TSSCP-P Br; aplicar os cuidados com base no TSSCP-P Br e realizar o preenchimento do TSSCP-P Br.

Os profissionais receberam por e-mail o link para acesso ao questionário de viabilidade disponível na plataforma *Google Forms*^®^, imediatamente após o término do período de aplicação do TSSCP-P Br.

### Análise dos dados

Para as variáveis quantitativas utilizou-se estatística descritiva, em números absolutos e relativos. Determinado a priori que a intervenção seria considerada viável e aceitável se os indicadores alcançassem um resultado > 80%^(16)^.

Na avaliação da viabilidade pelas mulheres sobreviventes foram considerados a taxa de retenção (nº de participantes presentes nos três tempos da coleta de dados do estudo experimental x 100/nº participantes que aceitaram participar), taxa de adesão (nº de tempos concluídos x 100/nº total de tempos)^(14)^. Para avaliar a viabilidade na perspectiva dos enfermeiros e a aceitabilidade das mulheres sobreviventes foram consideradas as opções favoráveis na escala de Likert (4 e 5 quando o esperado era concordância e 1 e 2 quando o esperado era discordância).

Para os dados qualitativos relacionados à percepção das mulheres sobreviventes de câncer de mama e profissionais sobre o TSSCP-P Br utilizou-se a análise de conteúdo de Bardin, seguindo os passos estruturados, compostos por: pré-análise, exploração do material e tratamento dos resultados, a inferência e a interpretação^(19)^. Os dados gerados foram selecionados, analisados e categorizados com o apoio do *software* ATLAS.ti 8.0 (*Scientific Software Development*, Berlin, Germany). A interpretação dos dados apoiou-se nas bases conceituais e operacionais das intervenções complexas de Sidani e Braden^(15)^.

### Aspectos éticos

O estudo obteve a aprovação dos Comitês de Ética em Pesquisa da Universidade Federal de São Paulo e Fundação Antônio Prudente, protocolo número 3.203.556/2019 e 3.351.638/2019. Todos os participantes forneceram consentimento informado.

## Resultados

Participaram do estudo 50 mulheres, todas vinculadas ao Sistema de Saúde Suplementar, e dez enfermeiros. Para melhor compreensão dos achados, inicialmente encontram-se apresentados os dados do estudo de aceitabilidade e viabilidade na perspectiva das mulheres sobreviventes e, na sequência, o de viabilidade na ótica dos profissionais.

Os dados sociodemográficos mostram que a média de idade das participantes foi de 55,7 anos (desvio-padrão de 12,1 anos), variando entre 36 e 81 anos. A maioria (66,0%) era casada ou vivia com um parceiro, 74,0% possuíam nível superior, e 60,0% identificaram-se como católicas. Em relação à classificação socioeconômica, 60,0% pertenciam à classe A e 32,0% à classe B. Quanto aos dados clínicos, a maioria das participantes apresentava carcinoma invasivo de tipo não-especial (72,0%), com estadiamento patológico I (34,0%) e II (32,0%). Os tratamentos mais comuns incluíram a combinação de cirurgia e radioterapia (54,0%) e de cirurgia, quimioterapia antineoplásica e radioterapia (32,0%).

A análise de viabilidade revelou uma taxa de retenção de 98,0%, abrangendo 50 das 51 participantes que consentiram em integrar o estudo; e a adesão atingiu 99,3% nos três tempos de coleta de dados relativos ao estudo experimental.

Em relação à aceitabilidade, observou-se uma taxa geral de 81,6%, com 93,3% para adequação; 85,0% para conveniência; 73,5% para eficácia e; 74,0% para adesão (Tabela 1).

**Tabela 1 -:** Aceitabilidade do TSSCP-P Br* por mulheres sobreviventes de câncer de mama (n^†^= 50). São Paulo, SP, Brasil, 2022

**Dimensão**	**Afirmativa**	**Discordo totalmente**	**Discordo**	**Neutro**	**Concordo**	**Concordo totalmente**
		**n** ^†^ **(%)**	**n** ^†^ **(%)**	**n** ^†^ **(%)**	**n** ^†^ **(%)**	**n** ^†^ **(%)**
Adequação	As orientações fornecidas pelo profissional foram adequadas.	0 (0,0)	0 (0,0)	2 (4,0)	11 (22,0)	37 (74,0)
	As informações contidas no plano de cuidados são claras e de fácil compreensão.	0 (0,0)	0 (0,0)	3 (6,0)	16 (32,0)	31 (62,0)
	O tamanho e tipo de letra, além da apresentação no formato impresso e modelo espiral, estão adequados.	0 (0,0)	0 (0,0)	2 (4,0)	11 (22,0)	37 (74,0)
Conveniência	As recomendações e orientações são fáceis de executar.	0 (0,0)	0 (0,0)	5 (10,0)	11 (22,0)	34 (68,0)
	O plano continha informações novas para você.	2 (4,0)	1 (2,0)	7 (14,0)	18 (36,0)	22 (44,0)
Eficácia	As informações contidas no plano podem trazer benefícios para a sua saúde e bem-estar.	0 (0,0)	0 (0,0)	2 (4,0)	15 (30,0)	33 (66,0)
	As informações contidas no plano de cuidados propiciaram alguma tomada de decisão para planejar os cuidados de seguimento adequados para você.	0 (0,0)	0 (0,0)	6 (12,0)	21 (42,0)	23 (46,0)
	Você se sente segura em seguir as recomendações contidas no plano de cuidados.	0 (0,0)	0 (0,0)	2 (4,0)	13 (26,0)	35 (70,0)
	Você se sentiu assustada ou desconfortável ao realizar a leitura do plano de cuidados.	22 (44,0)	12 (24,0)	9 (18,0)	4 (8,0)	3 (6,0)
Adesão	Você adotará o plano de cuidados como um guia de curto e longo prazo, para dar seguimento à sua saúde e bem-estar.	1 (2,0)	0 (0,0)	4 (8,0)	17 (34,0)	28 (56,0)
	Alguma sessão/parte do plano de cuidados não lhe parece útil.	16 (32,0)	13 (26,0)	9 (18,0)	8 (16,0)	4 (8,0)

*TSSCP-P Br = Resumo do Tratamento e Plano de Cuidados para a Sobrevivente de Câncer de Mama; ^†^n = Número de participantes

Das participantes, 39 (78,0%) responderam uma das duas perguntas que, recordando, consistiam em questionar sobre os tópicos interessantes, positivos e modificáveis, e se desejavam escrever comentários.

Procedeu-se à leitura e à organização dos dados de acordo com a natureza dos conteúdos por similaridade semântica, realizou-se a interpretação inferencial e contabilizou-se a frequência das unidades de registro. Os conteúdos de citações correspondentes às análises inferenciais e seus respectivos códigos referentes à percepção das mulheres sobre o TSSCP-P Br estão discriminados na Figura 1.

A avaliação da viabilidade do TSSCP-P Br na perspectiva do profissional teve a participação de dez enfermeiros, com média de idade de 30,6 anos (mínimo 23 e máximo 50), experiência em oncologia com média de 5,6 anos, variação de um a 21 anos, 60,0% com pós-graduação e 40,0% cursando a residência em oncologia.

Alcançou-se viabilidade geral de 84,2%, com 100,0% para adequação; 95,0% para conveniência; 100,0% para eficácia; 56,7% para riscos; 70% para adesão, 70% para disponibilidade, quantidade e habilidade de recursos humanos; 90% para recursos materiais, tecnológico e físico; 97,5% para fidelidade e 90% para alcance (Tabela 2).

**Figura 1 –:** Conteúdos de citações correspondentes às análises inferenciais e seus respectivos códigos referente a percepção das mulheres sobre o TSSCP-P Br*. São Paulo, SP, Brasil, 2022

**ID**	**Conteúdo de citação**	**Análise inferencial**	**Categorias**
17:2; 19:4; 20:2; 24:1; 25:2; 26:2; 30:4; 31:2; 38:2; 39:3; 40:3; 42:1; 45:2; 47:3; 49:2; 57:1	*Nada a acrescentar.*	Validação absoluta da apresentação e do conteúdo	Aceitabilidade e viabilidade asseguradas
55:1	*Eu não modificaria nada, está muito bem elaborado.*		
48:3	*Está muito bem elaborado.*		
34:1; 36:3; 48:2	*Não mudaria nada.*		
22:2	*Utilizei como guia orientador sobre meu tratamento (resumo de todo processo nas consultas médicas).*	Norteador e Guia	Viabilidade: orienta e favorece autogestão
46:2	*É muito útil no pós-tratamento para se adaptar ao novo normal. São muitos especialidades e prazos que não podemos perder.*		
37:1	*[...] auxiliar e orientar as pacientes durante o tratamento.*		
42:2	*Aplicação e diretriz.*		
21:1	*Pude esclarecer melhor os cuidados e conhecer o que estava acontecendo comigo.*		
47:2	*[...] dicas que são dadas pós-cirurgia também são muito importantes.*	Longitudinal e completo	Aceitabilidade: sumariza informações importantes
47:2	*[...] dicas que são dadas pós-cirurgia também são muito importantes.*		
49:1	*[...] interesse de melhorar o atendimento e o acompanhamento não só da doença em si, mas também do nosso psicológico.*		
16:3	*[...] todas as informações importantes sobre o tratamento em um único lugar.*		
30:2	*Muitos esclarecimentos.*		
35:1	*[...] informações são de extrema importância.*		
40:1	*[...] informações importantes para o tratamento.*		
30:2	*Muitos esclarecimentos.*		
26:1	*[...] informações sobre o meu tratamento (ponto positivo)*		
28:1	*[...] ter o histórico completo do meu diagnóstico (ponto positivo).*		
47:1	*Achei interessante o relato de todo o meu histórico desde o dia da cirurgia.*		
29:2	*Achei um material de qualidade, informativo e prático.*		
36:2	*[...] cuidado e aplicabilidade no dia a dia.*		
39:1	*[...] deixar claro que é possível ter uma vida após o câncer, se sentir mulher.*	Sentimentos positivos: conhecimento, segurança, autoconfiança, reconhecimento da iniciativa, da necessidade de ser um direito	Aceitabilidade: valoroso
39:2	*[...] o plano me fez bem.*		
39:4	*Continuem fazendo este trabalho. Depois de receber o plano fiz um ensaio fotográfico.*		
58:1	*Muito válida a orientação para seguir a vida com confiança e alegria.*		
45:1	*[...] conteúdo ajuda muito.*		
47:4	*Parabéns pelo excelente trabalho e pelo carinho.*		
45:3	*Achei a ideia muito boa, sugiro que continue.*		
56:1	*[...] sobrevivente de câncer é muito forte.*		
42:3	*Todos com câncer deveriam receber o manual.*		
39:1	*[...] deixar claro que é possível ter uma vida após o câncer, se sentir mulher.*		
32:2	*[...] gostei.*		
33:2	*[...] segurança.*	Sentimento positivo: segurança	
18:2	*Muitas coisas a serem aprendidas.*	Possibilita o aprendizado	Aceitabilidade: educativo
23:1	*Gostei das organizações e dos esclarecimentos.*		
53:2	*Me fez aprender.*		
25:1	*[...] esclarecimento da conduta e cuidados pós-tratamento.*		
54:1	*[...] melhora o conhecimento sobre sua doença.*		
36:1	*Linguagem de fácil entendimento.*	Conteúdo e linguagem adequados	Viabilidade: didático
22:1	*Organização do material e da forma como orienta.*		
38:1	*Leitura fácil, textos objetivos.*		
40:2	*[...] informações claras.*		
46:1	*A disposição dos assuntos está de fácil uso.*		
21:2	*Linguagem clara dos termos (ponto positivo).*		
46:1	*A disposição dos assuntos está de fácil uso.*		
17:1	*[...] esclarecimentos são objetivos.*	Além do biológico	Aceitabilidade: holístico
41:1	*[...] não me recordo de tudo que li.*		
43:1	*Ponto positivo: saúde e bem-estar.*		
20:1	*[...] cuidados com a mente.*		
41:2	*[...] ótimo para quem está no início do tratamento.*	Melhor aplicar no início do tratamento	Aceitabilidade e viabilidade relativizada: antecipar a entrega
41:3	*[...] superficial para quem está em um estágio do tamoxifeno.*	Superficial em alguns aspectos	Aceitabilidade e viabilidade relativizada: incompleto
41:4	*[...] pandemia e crise de gripe e esse controle da saúde também poderia conter no manual.*		
54:2	*Ter cartilha adequada para cada caso.*	Personalização/ Individualização	Aceitabilidade e viabilidade relativizada: individualizar
28:2	*Não usaria a palavra sobrevivente.*	Incômodo com o termo “sobrevivente”	Aceitabilidade e viabilidade relativizada: sobrevivente como denominação
56:1	*Sobrevivente de câncer é muito forte.*		
48:1	*Não li o plano porque não gosto de ler e também não gosto de lembrar da minha doença.*	Quantidade excessiva de conteúdos	Aceitabilidade e viabilidade relativizada: extenso

*TSSCP-P Br = Resumo do Tratamento e Plano de Cuidados para a Sobrevivente de Câncer de Mama

**Tabela 2 –:** Avaliação da aceitabilidade e viabilidade do TSSCP Br*, na perspectiva dos profissionais (n^†^ = 10). São Paulo, SP, Brasil, 2022

**Dimensão**	**Afirmativa**	**Avaliação**				
		**Discordo totalmente**	**Discordo**	**Neutro**	**Concordo**	**Concordo totalmente**
		**n** ^†^ **(%)**	**n** ^†^ **(%)**	**n** ^†^ **(%)**	**n** ^†^ **(%)**	**n** ^†^ **(%)**
Adequação	Os objetivos da intervenção correspondem aos priorizados para as mulheres sobreviventes de câncer de mama.	0 (0,0)	0 (0,0)	0 (0,0)	3 (30,0)	7 (70,0)
	As recomendações e orientações são aplicáveis à população de sobreviventes de câncer de mama.	0 (0,0)	0 (0,0)	0 (0,0)	3 (30,0)	7 (70,0)
	A forma como a intervenção está sendo implementada é capaz de gerar os objetivos almejados.	0 (0,0)	0 (0,0)	0 (0,0)	3 (30,0)	7 (70,0)
Conveniência	A intervenção é fácil de implementar na prática cotidiana.	0 (0,0)	1 (10,0)	0 (0,0)	4 (40,0)	5 (50,0)
	A equipe requer treinamento específico para realizar a intervenção.	0 (0,0)	0 (0,0)	0 (0,0)	4 (40,0)	6 (60,0)
Eficácia	A intervenção é eficaz, tanto no curto quanto no longo prazo.	0 (0,0)	0 (0,0)	0 (0,0)	4 (40,0)	6 (60,0)
Riscos	A intervenção pode causar dano ou desconforto para a sobrevivente de câncer de mama.	5 (50,0)	2 (20,0)	2 (20,0)	1 (10,0)	0 (0,0)
	A intervenção causa risco para o profissional da saúde.	9 (90,0)	1 (10,0)	0 (0,0)	0 (0,0)	0 (0,0)
Adesão	A sobrevivente de câncer de mama fará uso do material para melhorar a vigilância e os cuidados relacionados à saúde.	0 (0,0)	0 (0,0)	1 (10,0)	4 (40,0)	5 (50,0)
	A equipe de saúde adotará a intervenção no cotidiano de prática.	0 (0,0)	0 (0,0)	3 (30,0)	5 (50,0)	2 (20,0)
Disponibilidade, qualidade e habilidade dos recursos humanos	A intervenção requer recursos humanos adicionais.	0 (0,0)	2 (20,0)	3 (30,0)	3 (30,0)	2 (20,0)
	Existe profissionais disponíveis para aplicar a intervenção	0 (0,0)	1 (10,0)	1 (10,0)	7 (70,0)	1 (10,0)
	A intervenção requer recursos humanos especializados.	1 (10,0)	0 (0,0)	1 (10,0)	5 (50,0)	3 (30,0)
Treinamento	A equipe multiprofissional é capacitada para aplicar a intervenção.	0 (0,0)	2 (20,0)	0 (0,0)	4 (40,0)	4 (40,0)
Recursos materiais, tecnológicos e físico	O plano de cuidados no formato impresso é adequado.	0 (0,0)	1 (10,0)	0 (0,0)	1 (10,0)	8 (80,0)
	Os recursos tecnológicos para o acesso às informações sobre a intervenção são adequados.	0 (0,0)	0 (0,0)	0 (0,0)	3 (30,0)	7 (70,0)
	O ambiente físico para a realização da intervenção é adequado.	0 (0,0)	1 (10,0)	1 (10,0)	4 (40,0)	4 (40,0)
Fidelidade	O conteúdo contido no plano de cuidados é de fácil compreensão.	0 (0,0)	0 (0,0)	0 (0,0)	4 (40,0)	6 (60,0)
	As atividades relacionadas ao preenchimento do plano de cuidados são fáceis.	0 (0,0)	0 (0,0)	1 (10,0)	5 (50,0)	4 (40,0)
	As atividades relacionadas a aplicabilidade do plano são fáceis.	0 (0,0)	0 (0,0)	0 (0,0)	4 (40,0)	6 (60,0)
	O tempo para executar a intervenção, considerando o preenchimento e a aplicação é adequado.	0 (0,0)	0 (0,0)	0 (0,0)	7 (70,0)	3 (30,0)
Alcance	As atividades abrangem cuidados nas diversas fases de sobrevivência.	0 (0,0)	0 (0,0)	1 (10,0)	4 (40,0)	5 (50,0)

*TSSCP-P Br = Resumo do Tratamento e Plano de Cuidados para a Sobrevivente de Câncer de Mama; ^†^n = Número de participantes

Dos dez participantes, sete (70%) escreveram comentários sobre o TSSCP-P Br. Empregou-se a técnica de análise de conteúdo como no grupo de respondentes das mulheres sobreviventes. Os conteúdos de citações correspondentes às análises inferenciais e seus respectivos códigos, referente à percepção dos profissionais sobre o TSSCP-P Br estão discriminados na Figura 2.

**Figura 2 -:** Conteúdos de citações correspondentes às análises inferenciais e seus respectivos códigos, referentes à percepção dos profissionais sobre o TSSCP-P Br*. São Paulo, SP, Brasil, 2022

**ID**	**Conteúdo de citação**	**Análise inferencial**	**Categorias**
4:5	*[...] informações na palma da mão.*	Satisfação profissional	Aceitabilidade: contribui para o exercício profissional
4:3	*[...] tamanho de bolsa.*		
4:6	*[...] versão digital ou física.*		
1:1	*Foi uma experiência incrível poder contribuir além de aprender muito com todos*		
3:4	*[...] expressões de gratidão e acolhimento envolvendo família e a próprio paciente.*		
3:7	*Oportunidade de fazer parte desse processo.*		
4:1	*[...] prazeroso.*		
7:1	*Experiência pessoal e profissional muito especial.*		
2:5	*[...] um “elo” entre paciente e profissional.*		
3:3	*[...] plano incentiva questões de pergunta.*	Favorece atividade de trabalho	
5:3	*Acompanhamento do progresso do tratamento.*		
5:2	*[...] facilitador em consultas futuras.*		
2:1	*[...] uma grande ferramenta não só de informação (...)*		
3:5	*[...] nas pacientes que abordei(...) houve expressões de gratidão e acolhimento, envolvendo família e paciente.*	Qualidade no cuidado	Viabilidade: cuidado centrado no paciente e família
2:4	*[...] traz acolhimento e cuidado com a paciente.*		
6:1	*[...] o olhar do profissional da saúde para a sobrevivente que já passou pelo processo mais difícil da doença: descoberta, tratamento e sofrimento físico e social.*		
6:2	*[...] empático. Nós, profissionais, nos esquecemos que a vida da sobrevivente muda. É necessário dar orientações para levar a vida de outra maneira.*		
5:1	*Fundamental importância para a paciente, pois traz os dados desde o início do tratamento.*		
3:5	*[...] nas pacientes que abordei [...] houve expressões de gratidão e acolhimento, envolvendo família e paciente.*		Viabilidade: considera as implicações da doença oncológica na sobrevivente
4:4	*[...] forma de* app *.*	Com uso de tecnologia digital	Aceitabilidade relativizada: forma de apresentação
4:5	*Informações na palma da mão.*	Apresentação compactada	
4:3	*[...] tamanho de bolsa.*		
4:6	*Versão digital ou física.*	Com mais de uma apresentação	
4:2	*O Plano contém todas as informações, porém, poderia ser mais compacto.*	Completo, com potencial para ser resumido	Viabilidade relativizada: excesso de conteúdos
3:1	*[...] duas pacientes se sentiram desconfortáveis com a palavra sobrevivente*	Palavra sobrevivente pode gerar desconforto na paciente	Viabilidade relativizada: sobrevivente como denominação

*TSSCP-P Br = Resumo do Tratamento e Plano de Cuidados para a Sobrevivente de Câncer de Mama

## Discussão

O estudo apresentou dados que reforçam a aceitação e viabilidade do TSSCP-P Br como recurso viável e qualificador da assistência de mulheres sobreviventes de câncer de mama. A maioria relatou que o plano continha informações novas, que as informações contidas nele propiciaram alguma tomada de decisão para planejar os cuidados de seguimento e que as recomendações e orientações eram fáceis de executar. Em um estudo que avaliou o impacto do fornecimento de planos de cuidados para sobreviventes, também identificou-se que a maioria dos participantes consideraram as informações do plano como novas^(9,20)^.

Sobreviventes de câncer valorizam intervenções com conteúdo específico para suas necessidades, que sejam fáceis de utilizar, acessíveis e entregues no momento adequado durante a jornada do câncer^(21)^. É importante salientar que as necessidades e preferências variam de pessoa para pessoa em todas as fases da jornada, incluindo o período pós-tratamento, e que todos os sobreviventes precisam de informações abrangentes e relevantes para orientá-los na gestão da saúde^(7,20)^.

Conforme constatado neste estudo, o TSSCP-P Br pode ser adotado como um guia de grande utilidade em todas as suas partes constituintes, no curto e longo prazo da sobrevivência, para dar seguimento à saúde e bem-estar. Os dados corroboram estudos que indicam que a melhoria do conhecimento do paciente sobre sua doença e tratamento gera maior engajamento com o processo de gestão da saúde^(22-23)^. Isso também se alinhou aos resultados satisfatórios em termos de viabilidade, com alta adesão e retenção, que demonstrou maior participação no processo de autogestão da saúde.

Para os enfermeiros envolvidos na implementação, o TSSCP-P Br foi considerado viável, embora alguns fatores limitantes tenham sido apontados, especialmente em relação ao quantitativo de profissionais e à especificidade técnica destes na área de oncologia. A necessidade de recursos humanos complementares pode ser justificada pelo tempo necessário para a entrega do plano, que pode variar de 20 a 90 minutos de acordo com a literatura^(20)^. Além disso, a formação inadequada dos profissionais para o cuidado de sobreviventes de câncer também é um fator limitante amplamente discutido e que pode impactar negativamente na prática clínica^(20)^. É importante destacar que a percepção dos profissionais ao aplicar uma intervenção é influenciada por diversos fatores, como valores e crenças pessoais, formação profissional, conhecimento teórico, experiência prática e o uso de diretrizes de boas práticas^(16)^.

Quando os profissionais consideram uma intervenção inaceitável para os pacientes, é possível evitá-la, portanto, a percepção sobre a habilidade em executá-la assim como a praticidade na aplicação podem aumentar a motivação para executar a intervenção, impactando na fidelidade para a adoção das ações contidas no plano^(17)^.

A implementação de intervenções exitosas advém da aceitabilidade por parte dos usuários. Aceitabilidade é um construto multifacetado que reflete até que ponto as pessoas que realizam ou recebem uma intervenção de saúde a consideram apropriada. O referencial teórico da aceitabilidade é composto por sete componentes: atitude afetiva, sobrecarga, aplicabilidade, ética, coerência da intervenção, custos e autoeficácia^(12)^.

As possibilidades analíticas dos depoimentos evidenciaram que as respostas discursivas foram extremamente esclarecedoras, pois revelaram percepções que não puderam ser exploradas nas respostas em escala de *Likert*, aumentando a validade dos achados^(9,15)^.

Na análise qualitativa dos depoimentos sobre os atributos do TSSCP-P Br, na perspectiva das mulheres sobreviventes de câncer de mama, foram geradas categorias que expressaram várias qualidades do instrumento. Essa população valoriza o acesso a informações de autocuidado organizadas com base no *continuum* do câncer, de forma a apoiar o autogerenciamento^(24)^.

Do ponto de vista dos profissionais que aplicaram a intervenção, vários aspectos positivos também foram destacados, principalmente como uma possibilidade de materialização do cuidado centrado no paciente. Em uma pesquisa, que objetivou desenvolver e avaliar o TSSCP-S, os avaliadores (profissionais e mulheres sobreviventes de câncer de mama) observaram que o seu uso propiciou o cuidado centrado no paciente^(25)^.

O cuidado centrado na pessoa está entre os principais modelos que contribuem para a excelência da assistência^(26)^, defendido por instituições reguladoras em saúde e por aquelas que possuem programas de acreditação hospitalar, como o Sistema Brasileiro de Acreditação (SBA-ONA), o da *Joint Commission International* (JCI), baseada no modelo Norte-Americano; e a da Acreditação Canadense (*Accreditation Canada International* - ACI), recentemente renomeada como *Health Standards Organization* (HSO)^(27)^.

Há de se destacar que a instituição sede do estudo é acreditada pela *Accreditation Canada International* e isso significa que os resultados desta pesquisa, ao serem incorporados no cotidiano de prática, impulsionarão as conquistas para outros patamares, ainda superiores.

Ademais, para ambos os respondentes, aspectos negativos do TSSCP-P Br foram desvelados, como possível sofrimento gerado por informações que antecipam fatos e riscos, a generalidade e extensão e o emprego do termo “sobrevivente”. O termo “sobrevivente de câncer” é amplamente utilizado por diferentes pessoas, instituições de saúde, órgãos acadêmicos e organizações políticas. No entanto, em muitos países, os pacientes interpretam os termos de forma negativa, associando-os à lembrança do alto risco de morte e à dissociação com a cura, o que gera rejeição, pois continuam lidando com o medo de recorrência^(28)^.

Comparando os achados com a literatura, uma meta-análise que examinou a viabilidade da implementação de planos de cuidados para sobreviventes de câncer, sob as perspectivas dos sobreviventes e profissionais de saúde, concluiu que os planos são aceitáveis e valorizados por ambos^(20)^. Enfim, avaliar a aceitabilidade e viabilidade pode auxiliar na identificação de facilitadores e barreiras para a implementação da intervenção e compreender o alcance dos resultados esperados, como atestado em vasta literatura sobre o tema^(16,29)^.

Dentre as limitações, destaca-se a coleta em sede única, além do fato de a instituição em questão se encontrar na definição de um *cancer center*, com acreditação hospitalar. Infelizmente, esta não é a realidade da maioria dos centros de tratamento públicos do câncer no Brasil, que se encontram em hospitais gerais, não acreditados em Oncologia, com um número limitado de profissionais especializados e estruturas físicas deficientes para a assistência de excelência.

Outra importante limitação é a caracterização sociodemográfica das sobreviventes de câncer de mama que apresentam um perfil distinto do cenário nacional, dificultando a generalização dos resultados para as sobreviventes exclusivas do sistema público de saúde brasileiro. Um número amostral maior de profissionais também deve ser considerado em estudo futuro para confirmação dos achados.

Na perspectiva das contribuições do presente estudo para o aprimoramento da ciência da enfermagem, cabe destacar a importância da realização de estudos que objetivam avaliar aceitabilidade e viabilidade de propostas inovadoras. O estudo de aceitabilidade e viabilidade acrescentou, de fato, um conjunto de dados que pode favorecer o refinamento da aplicação do TSSCP-P Br no cuidado de mulheres sobreviventes de câncer de mama após o término do tratamento inicial.

## Conclusão

Os dados quantitativos deste estudo indicaram que o TSSCP-P Br alcançou níveis satisfatórios de aceitabilidade e viabilidade satisfatórias. Do ponto de vista das mulheres sobreviventes de câncer de mama, a viabilidade apresentou valores elevados para retenção e adesão, bem como para a aceitabilidade em todas as dimensões. Na perspectiva dos profissionais, a aceitabilidade e viabilidade também mostraram valores elevados em quase todas as dimensões avaliadas.

Na análise qualitativa, as mulheres sobreviventes de câncer de mama elogiaram o TSSCP-P Br, evidenciando o quanto é valoroso, educativo, capaz de sumarizar informações importantes, favorecer a autogestão e ser didático. Mas também, com o risco de ser incompleto ou extenso, além de manifestarem desconforto com o uso do termo “sobrevivente”.

A avaliação dos enfermeiros sobre TSSCP-P Br também foi positiva por favorecer o exercício profissional qualificado, o cuidado centrado no paciente e na família, e ser capaz de reunir a complexidade da doença oncológica no contexto da sobrevivência. No entanto, os profissionais sugeriram a possibilidade de compactar conteúdos, disponibilizá-lo no formato digital e alertaram para o risco de o termo “sobrevivente” gerar desconforto nas pacientes.

# Translation: Resumen del Tratamiento y Plan de Cuidados para Sobrevivientes de Cáncer de Mama: estudio de aceptabilidad y viabilidad*

**DOI:** 10.1590/1518-8345.7476.4619

**Authors:** Maria das Graças Silva Matsubara, Fabiana Baroni Alves Makdissi, Simone Elias, Cristiane Decat Bergerot, Kimlin Tan Ashing, Edvane Birelo Lopes De Domenico

**Affiliations:** 1 AC Camargo Cancer Center, São Paulo, SP, Brasil.; 2 Univerdade Federal de São Paulo, São Paulo, SP, Brasil.; 3 Oncoclínicas, Brasília, DF, Brasil.; 4 City of Hope National Medical Center, California, Duarte, CA, Estados Unidos da América.

**Keywords:** Neoplasias de la Mama, Supervivientes de Cáncer, Estudios de Factibilidad, Planificación de Atención al Paciente, Perfil de Impacto de Enfermedad, Oncología Médica

## Abstract

evaluar la aceptabilidad y viabilidad del documento Resumen del Tratamiento y Plan de Cuidados para la Sobreviviente de Cáncer de Mama (TSSCP-P Br).

estudio transversal, cuantitativo-cualitativo, con la participación de mujeres que finalizaron el tratamiento para el cáncer de mama (n=50) y enfermeros (n=10) que incorporaron el documento al plan de cuidados en las consultas de seguimiento ambulatorio durante la fase experimental de un estudio clínico. Los cuestionarios de viabilidad y aceptabilidad se aplicaron en la última etapa de recolección de datos del experimento. Los datos fueron analizados mediante estadística descriptiva y análisis de contenido.

en la evaluación de las mujeres sobrevivientes, la viabilidad demostró una retención del 98,0% y una adhesión del 99,3%. La aceptabilidad en cuanto a adecuación, conveniencia, eficacia y adhesión alcanzó el 81,6%. Desde la perspectiva de los profesionales, la viabilidad y la aceptabilidad fueron del 84,2%, en cuanto a adecuación, conveniencia, eficacia, riesgos, disponibilidad, capacitación, fidelidad, alcance y recursos. Se ensalzó el documento y se señalaron los principales problemas de la práctica, como la experiencia del profesional.

el Resumen del Tratamiento y Plan de Cuidados demostró ser viable y aceptable para la práctica clínica del cuidado de mujeres sobrevivientes de cáncer de mama.

## Destacados

**(1)** El Plan de Cuidados contribuye a la autogestión del sobreviviente de cáncer. **(2)** El Plan de Cuidados promueve la atención centrada en la persona sobreviviente de cáncer. **(3)** Los sobrevivientes de cáncer evaluaron el Plan de Cuidados como un derecho postratamiento. **(4)** Los enfermeros consideraron el Plan de Cuidados como adecuado, conveniente y eficaz. **(5)** La implementación del Plan de Cuidados exige competencias específicas en oncología.

## Introducción

El cáncer de mama es el más frecuente entre las mujeres en todo el mundo, con más de 2 millones de diagnósticos anuales y, en Brasil, con 73 mil nuevos casos estimados para el trienio 2023-2025^(1-2)^.

Se prevé que estos números aumenten debido al envejecimiento de la población y a las tendencias crecientes de los factores de riesgo modificables del cáncer de mama, lo que, en consecuencia, resultará en un aumento de la población de sobrevivientes, especialmente a raíz de los diagnósticos precoces y tratamientos combinados eficaces^(1)^. Actualmente, las tasas globales de supervivencia a 5 años varían en más de 90% en países desarrollados y del 40 al 66% en países de renta media y baja, respectivamente^(3)^.

Este escenario epidemiológico ha resultado en nuevos desafíos para brindar la mejor atención posible a la creciente población de sobrevivientes^(4)^. Conceptualmente, el sobreviviente de cáncer es la persona que ha tenido cáncer y que se encuentra en el recorrido desde el diagnóstico hasta el tratamiento o más allá, a lo largo de la vida^(5)^.

En la condición de sobreviviente a largo plazo, existe la falsa percepción de que, al término del tratamiento, el sobreviviente presentará una mejora global de los signos y síntomas con el paso del tiempo. Sin embargo, los sobrevivientes de cáncer de mama pueden experimentar complicaciones tardías y a largo plazo debido a la enfermedad y a los tratamientos, cirugía, quimioterapia antineoplásica y/o radioterapia. Estos efectos adversos pueden resultar en problemas relacionados con la salud mental, física y social, que culminan en el deterioro de la calidad de vida (CV)^(6)^.

Así, para mejorar la CV se deben proporcionar intervenciones adecuadas a las necesidades de salud, incluyendo orientaciones sobre el manejo de los efectos secundarios a largo plazo, señales de recurrencia, salud mental, consultas de seguimiento y estilo de vida saludable^(7)^. Sin embargo, muchos sobrevivientes se encuentran desprovistos de información completa y segura sobre la enfermedad oncológica ante la necesidad de comprender las manifestaciones tardías que presenta y de reportar el recorrido para que los profesionales puedan tratar los efectos adversos^(8)^.

La *National Coalition for Cancer Survivorship* (NCCS), una organización sin fines de lucro liderada por sobrevivientes de cáncer de América, que tiene como objetivo defender cuidados de calidad para las personas con cáncer, afirma que todos los sobrevivientes de cáncer deben tener un resumen de tratamiento y plan de cuidados que atienda las necesidades postratamiento para mejorar la salud y la CV, tan pronto como se finalice el tratamiento inicial del cáncer^(5)^. Diversos estudios ya se han realizado a partir del plan de cuidados para sobrevivientes de cáncer e indicaron las potencialidades del recurso después del término del tratamiento inicial^(7,9-10)^.

Así, se obtuvo una versión nacional a partir de la traducción y adaptación transcultural del *Treatment Summary and Survivorship Care Plan* (TSSCP-S) para sobrevivientes brasileñas de cáncer de mama, resultando en el Resumen del Tratamiento y Plan de Cuidados para la Sobreviviente de Cáncer de Mama (TSSCP-P Br), compuesto por: qué es un plan de cuidados para la supervivencia, su importancia, cómo utilizarlo, información sobre el cáncer de mama, datos sobre el diagnóstico y tratamiento del cáncer, cuidados de seguimiento y vigilancia, equipo de cuidados, consejos de salud y cuestiones sobre CV^(11)^.

El TSSCP-P Br demostró un impacto positivo en la autoeficacia, bienestar físico y emocional de las sobrevivientes de cáncer de mama^(12)^. En este estudio brasileño se buscó, al término de la intervención, evaluar la aceptabilidad y viabilidad desde la perspectiva tanto de los profesionales como de los receptores de la intervención, para estimar el potencial de su aplicación en el mundo real, además de posibilitar ajustes en estudios y prácticas futuras; siendo estos los datos presentados en la presente investigación.

Actualmente, se reconoce cada vez más que la aceptabilidad y la viabilidad deben ser consideradas al concebir, evaluar e implementar intervenciones de salud^(13-14)^. La viabilidad confirma si la intervención puede implementarse según lo planeado, mientras que la aceptabilidad indica si los destinatarios potenciales están dispuestos y son capaces de recibir y adherirse a la intervención^(13,15)^.

Existe una tendencia global de búsqueda de evidencias sobre la capacidad de implementación de planes de cuidados para sobrevivientes de cáncer^(9)^ que justifica la intencionalidad del presente estudio. Así, la aceptabilidad, en la presente investigación, se refirió a la forma en que las pacientes y los profesionales de salud recibieron y utilizaron el TSSCP-P Br, mientras que la viabilidad se relacionó con las potencialidades de implementación del plan en la práctica y su capacidad de apoyar la continuidad del cuidado. Los objetivos fueron evaluar la aceptabilidad y viabilidad del documento Resumen del Tratamiento y Plan de Cuidados para la Sobreviviente de Cáncer de Mama (TSSCP-P Br).

## Método

### Diseño del estudio

Estudio transversal, cuantitativo-cualitativo, parte integrante de una extensa investigación^(11-12)^. La presente investigación utilizó directrices ampliamente recomendadas para estudios que pretenden evaluar aceptabilidad y viabilidad^(13,15-16)^ y de acuerdo con la herramienta *Revised Standards for Quality Improvement Reporting Excellence* (SQUIRE 2.0).

### Ubicación y período

El estudio se realizó en un *Cancer Center* de la ciudad de São Paulo, SP, Brasil, en el período de junio de 2021 a mayo de 2022. La institución sede es filantrópica, con asistencia vinculada al *Sistema Único de Saúde*, de acceso gratuito y universal, y de Salud Suplementaria, vinculada a las empresas proveedoras de servicios de salud.

### Población y criterios de selección y definición de los participantes

Los participantes de este estudio fueron 50 mujeres sobrevivientes de cáncer de mama, de las 51 que consintieron participar y recibieron el TSSCP-P Br, además de 10 enfermeras que orientaron sobre su finalidad y uso. Los criterios de inclusión para la selección de las pacientes fueron: pertenecer al grupo experimental, edad mayor de 18 años, sexo femenino, con diagnóstico de cáncer de mama, en cualquier estadificación patológica, sometidas a terapias clínicas con quimioterapia antineoplásica, radioterapia y tratamiento quirúrgico, atendidas exclusivamente en la institución sede del estudio, que finalizaron el tratamiento (excepto terapia endocrina). Se excluyeron las pacientes que no se sometieron a procedimientos quirúrgicos para el tratamiento del cáncer de mama; con historial de otros cánceres, excepto cáncer de piel no melanoma; que no fueran fluidas en el idioma portugués y portadoras de trastornos psiquiátricos registrados en la historia clínica.

En relación con las enfermeras que participaron en la intervención, estas fueron reclutadas del Programa de Residencia Multiprofesional y de la Educación Permanente de la institución sede del estudio. Para ser elegibles, debían poseer posgrado en oncología y/o estar cursando la especialización en la modalidad de residencia; declarar disponibilidad y participar en el entrenamiento para la aplicación del TSSCP-P Br.

En relación con el cálculo muestral, en la literatura especializada se evidencia que un mínimo de 30 participantes se considera adecuado para estudios que pretenden evaluar si una intervención es apropiada^(16-17)^.

### Variables del estudio

Las variables dependientes fueron la aceptabilidad y la viabilidad del TSSCP-P Br, mientras que las variables independientes incluyeron los datos sociodemográficos (edad, estado civil, nivel de instrucción, creencia religiosa, clasificación socioeconómica y experiencia en oncología) y clínicos (tipo histológico del cáncer de mama, estadificación y tratamiento).

### Instrumentos utilizados para la recolección de la información

Los instrumentos para evaluar la aceptabilidad por parte de las pacientes y los profesionales se elaboraron basándose en los preceptos de las intervenciones complejas en salud de Sidani y Braden^(15)^, así como en el marco teórico de aceptabilidad de Sekhon, et al.^(14)^, optándose por los criterios comunes y aquellos estrechamente relacionados con los objetivos de la presente investigación.

El instrumento destinado a la evaluación de la viabilidad por parte de los profesionales se fundamentó en el *Structured Assessment of Feasibility* (SAFE), una medida estandarizada para evaluar la viabilidad de implementación de intervenciones complejas en los servicios de salud mental del *National Health Service* (NHS), pero que puede aplicarse en una variedad de estudios, desde intervenciones farmacológicas simples hasta innovaciones complejas institucionales, sin necesidad de obtener autorización, siempre que se cite debidamente, conforme a lo orientado por los autores^(18)^ (cuestionarios disponibles en: https://bit.ly/3wXe0UU).

La evaluación de la viabilidad por parte de las pacientes se realizó basándose en las tasas de adhesión y retención^(14)^.

El cuestionario destinado a la evaluación de la aceptabilidad por parte de las pacientes estuvo compuesto por cuatro dimensiones y 11 ítems: (1) Adecuación (tres ítems); (2) Conveniencia (dos ítems); (3) Eficacia (cuatro ítems); (4) Adhesión (dos ítems).

Al final del cuestionario había un espacio para comentarios, sugerencias y las siguientes preguntas: ¿Qué le pareció más interesante y que considera como punto positivo? ¿Qué modificaría?

Dado que las encuestadas escribieron de su puño y letra, la validación se realizó el día de la recolección de datos, buscando aclarar los contenidos elegibles y los de difícil interpretación, legitimando así las respuestas con las encuestadas.

El cuestionario para las evaluaciones de aceptabilidad y viabilidad por parte de las enfermeras estuvo compuesto por diez dimensiones y 22 ítems: (1) Adecuación (tres ítems); (2) Conveniencia (dos ítems); (3) Eficacia (un ítem); (4) Riesgos (dos ítems); (5) Adhesión (dos ítems); (6) Disponibilidad, cantidad y habilidad de los recursos humanos (tres ítems); (7) Capacitación (un ítem); (8) Recursos materiales, tecnológicos y físicos (tres ítems); (9) Fidelidad (cuatro ítems) y; (10) Alcance (un ítem). Al final del cuestionario había un espacio para comentarios y sugerencias. La validación del contenido relativo a los comentarios y sugerencias se realizó posteriormente mediante contacto vía correo electrónico.

Las evaluaciones de las dimensiones de ambos cuestionarios se realizaron mediante una escala tipo Likert de 5 puntos, siendo: 1. Totalmente en desacuerdo; 2. En desacuerdo; 3. Neutral; 4. De acuerdo; 5. Totalmente de acuerdo.

### Recolección de datos

La aplicación del TSSCP-P Br se realizó mediante dos encuentros individuales, con un intervalo de tres meses entre ellos y una duración que varió de 30 a 90 minutos. En el primer encuentro, las participantes recibieron el TSSCP-P Br en formato de folleto y visualizaron un video explicativo que abordaba el contenido del plan de cuidados. Las participantes también proporcionaron información demográfica, que no estaba incluida en el PEP. En el segundo encuentro, se aclararon dudas y se reforzaron las competencias adquiridas, además del desarrollo de nuevas habilidades para lidiar con los efectos tardíos, con una duración de 20 a 60 minutos^(12)^.

En el tercer y último encuentro, después del período de seis meses de prueba del TSSCP-P Br, se aplicó el cuestionario de aceptabilidad a las mujeres sobrevivientes de cáncer de mama que habían participado en los dos encuentros anteriores.

Las enfermeras participaron en un entrenamiento teórico-práctico, presencial, con una carga horaria de dos horas, realizado por la investigadora principal, en el cual se aclaró el TSSCP-P Br, y se discutieron y pusieron a disposición un guion y un video que contenían el enfoque a realizar con la paciente. Los objetivos de aprendizaje del entrenamiento fueron conocer los datos epidemiológicos, factores de riesgo, etiología y diagnóstico del cáncer de mama; comprender la clasificación, estadificación y tratamiento del cáncer de mama; entender el concepto de sobreviviente de cáncer y la importancia del plan de cuidados; conocer el TSSCP-P Br; aplicar los cuidados basados en el TSSCP-P Br y realizar el llenado del TSSCP-P Br.

Los profesionales recibieron por correo electrónico el enlace para acceder al cuestionario de viabilidad disponible en la plataforma *Google Forms* ^®^, inmediatamente después de concluir el período de aplicación del TSSCP-P Br.

### Análisis de los datos

Para las variables cuantitativas se utilizó estadística descriptiva, en números absolutos y relativos. Se determinó a *priori* que la intervención se consideraría viable y aceptable si los indicadores alcanzaban un resultado > 80%^(16)^.

En la evaluación de la viabilidad por parte de las mujeres sobrevivientes se consideraron la tasa de retención (número de participantes presentes en los tres momentos de recolección de datos del estudio experimental x 100/número de participantes que aceptaron participar) y la tasa de adhesión (número de momentos completados x 100/número total de momentos)^(14)^. Para evaluar la viabilidad desde la perspectiva de las enfermeras y la aceptabilidad de las mujeres sobrevivientes se consideraron las opciones favorables en la escala Likert (4 y 5 cuando se esperaba acuerdo y 1 y 2 cuando se esperaba desacuerdo).

Para los datos cualitativos relacionados con la percepción de las mujeres sobrevivientes de cáncer de mama y de los profesionales sobre el TSSCP-P Br se utilizó el análisis de contenido de Bardin, siguiendo los pasos estructurados, compuestos por: preanálisis, exploración del material y tratamiento de los resultados, inferencia e interpretación^(19)^. Los datos generados fueron seleccionados, analizados y categorizados con el apoyo del *software* ATLAS.ti 8.0 (*Scientific Software Development, Berlin, Germany*). La interpretación de los datos se apoyó en las bases conceptuales y operativas de las intervenciones complejas de Sidani y Braden^(15)^.

### Aspectos éticos

El estudio obtuvo la aprobación de los Comités de Ética en Investigación de la *Universidade Federal de São Paulo* y la *Fundação Antônio Prudente*, protocolo número 3.203.556/2019 y 3.351.638/2019. Todos los participantes proporcionaron su consentimiento informado.

## Resultados

Participaron en el estudio 50 mujeres, todas vinculadas al Sistema de Salud Suplementaria, y 10 enfermeros. Para una mejor comprensión de los hallazgos, se presentan inicialmente los datos del estudio de aceptabilidad y viabilidad desde la perspectiva de las mujeres sobrevivientes y, a continuación, la de viabilidad en la óptica de los profesionales.

Los datos sociodemográficos muestran que la media de edad de las participantes fue de 55,7 años (desviación estándar de 12,1 años), variando entre 36 y 81 años. La mayoría (66,0%) estaba casada o convivía con una pareja, 74,0% poseían nivel superior, y 60,0% se identificaron como católicas. En relación con la clasificación socioeconómica, 60,0% pertenecían a la clase A y 32,0% a la clase B. En cuanto a los datos clínicos, la mayoría de las participantes presentaba carcinoma invasivo de tipo no-especial (72,0%), con estadificación patológica I (34,0%) y II (32,0%). Los tratamientos más comunes incluyeron la combinación de cirugía y radioterapia (54,0%) y de cirugía, quimioterapia antineoplásica y radioterapia (32,0%).

El análisis de viabilidad reveló una tasa de retención de 98,0%, abarcando 50 de las 51 participantes que consintieron integrar el estudio; y la adhesión alcanzó 99,3% en los tres momentos de recolección de datos relativos al estudio experimental.

Con relación a la aceptabilidad se observó una tasa general de 81,6%, con 93,3% para adecuación; 85,0% para conveniencia; 73,5% para eficacia; y 74,0% para adhesión (Tabla 1).

**Tabla 1 -:** Aceptabilidad del TSSCP-P Br* por mujeres sobrevivientes de cáncer de mama (n^†^= 50). São Paulo, SP, Brasil, 2022

**Dimensión**	**Afirmativa**	**Totalmente en desacuerdo**	**En desacuerdo**	**Neutro**	**De acuerdo**	**Totalmente de acuerdo**
		**n** ^†^ **(%)**	**n** ^†^ **(%)**	**n** ^†^ **(%)**	**n** ^†^ **(%)**	**n** ^†^ **(%)**
Adecuación	Las orientaciones proporcionadas por el profesional fueron adecuadas.	0 (0,0)	0 (0,0)	2 (4,0)	11 (22,0)	37 (74,0)
	La información contenida en el plan de cuidados es clara y de fácil comprensión.	0 (0,0)	0 (0,0)	3 (6,0)	16 (32,0)	31 (62,0)
	El tamaño y tipo de letra, además de la presentación en formato impreso y modelo de espiral, están adecuados.	0 (0,0)	0 (0,0)	2 (4,0)	11 (22,0)	37 (74,0)
Conveniencia	Las recomendaciones y orientaciones son fáciles de ejecutar.	0 (0,0)	0 (0,0)	5 (10,0)	11 (22,0)	34 (68,0)
	El plan contenía información nueva para usted.	2 (4,0)	1 (2,0)	7 (14,0)	18 (36,0)	22 (44,0)
Eficacia	La información contenida en el plan puede aportar beneficios para su salud y bienestar.	0 (0,0)	0 (0,0)	2 (4,0)	15 (30,0)	33 (66,0)
	La información contenida en el plan de cuidados propició alguna toma de decisiones para planificar los cuidados de seguimiento adecuados para usted.	0 (0,0)	0 (0,0)	6 (12,0)	21 (42,0)	23 (46,0)
	Usted se siente segura al seguir las recomendaciones contenidas en el plan de cuidados.	0 (0,0)	0 (0,0)	2 (4,0)	13 (26,0)	35 (70,0)
	Usted se sintió asustada o incómoda al realizar la lectura del plan de cuidados.	22 (44,0)	12 (24,0)	9 (18,0)	4 (8,0)	3 (6,0)
Adhesión	Usted adoptará el plan de cuidados como una guía a corto y largo plazo, para dar seguimiento a su salud y bienestar.	1 (2,0)	0 (0,0)	4 (8,0)	17 (34,0)	28 (56,0)
	Alguna sesión/parte del plan de cuidados no le parece útil.	16 (32,0)	13 (26,0)	9 (18,0)	8 (16,0)	4 (8,0)

*TSSCP-P Br = Resumen del Tratamiento y Plan de Cuidados para la Sobreviviente de Cáncer de Mama; ^†^n = Número de participantes

De las participantes, 39 (78,0%) respondieron a una de las dos preguntas que, recordando, consistían en indagar sobre los temas interesantes, positivos y modificables, y si deseaban escribir comentarios.

Se procedió a la lectura y organización de los datos de acuerdo con la naturaleza de los contenidos por similitud semántica, se realizó la interpretación inferencial y se contabilizó la frecuencia de las unidades de registro. Los contenidos de las citas correspondientes a los análisis inferenciales y sus respectivos códigos referentes a la percepción de las mujeres sobre el TSSCP-P Br están discriminados en la Figura 1.

La evaluación de la viabilidad del TSSCP-P Br desde la perspectiva del profesional contó con la participación de diez enfermeros, con una media de edad de 30,6 años (mínimo 23 y máximo 50), experiencia en oncología con una media de 5,6 años, variación de uno a 21 años, 60,0% con posgrado y 40,0% cursando la residencia en oncología.

Se alcanzó una viabilidad general de 84,2%, con 100,0% para adecuación; 95,0% para conveniencia; 100,0% para eficacia; 56,7% para riesgos; 70% para adhesión, 70% para disponibilidad, cantidad y habilidad de recursos humanos; 90% para recursos materiales, tecnológicos y físicos; 97,5% para fidelidad y 90% para alcance (Tabla 2).

**Figura 1 –:** Contenidos de citas correspondientes a los análisis inferenciales y sus respectivos códigos referentes a la percepción de las mujeres sobre el TSSCP-P Br*. São Paulo, SP, Brasil, 2022

**ID**	**Contenido de cita**	**Análisis inferencial**	**Categorías**
17:2; 19:4; 20:2; 24:1; 25:2; 26:2; 30:4; 31:2; 38:2; 39:3; 40:3; 42:1; 45:2; 47:3; 49:2; 57:1	*Nada que añadir.*	Validación absoluta de la presentación y del contenido	Aceptabilidad y viabilidad aseguradas
55:1	*No modificaría nada, está muy bien elaborado.*		
48:3	*No modificaría nada, está muy bien elaborado.*		
34:1; 36:3; 48:2	*No cambiaría nada.*		
22:2	*Utilicé como guía orientadora sobre mi tratamiento (resumen de todo el proceso en las consultas médicas).*	Orientador y Guía	Viabilidad: orienta y favorece la autogestión
46:2	*Es muy útil en el postratamiento para adaptarse a la nueva normalidad. Son muchas especialidades y plazos que no podemos perder.*		
37:1	*[...] auxiliar y orientar a las pacientes durante el tratamiento.*		
42:2	*Aplicación y directriz.*		
21:1	*Pude aclarar mejor los cuidados y conocer lo que estaba sucediendo conmigo.*		
47:2	*[...] los consejos que se dan postcirugía también son muy importantes.*	Longitudinal y completo	Aceptabilidad: resume información importante
47:2	*[...] los consejos que se dan postcirugía también son muy importantes.*		
49:1	*[...] interés en mejorar la atención y el seguimiento no solo de la enfermedad en sí, sino también de nuestro psicológico.*		
16:3	*[...] toda la información importante sobre el tratamiento en un solo lugar.*		
30:2	*Muchos aclaraciones.*		
35:1	*[...] la información es de extrema importancia.*		
40:1	*[...] información importante para el tratamiento.*		
30:2	*Muchas aclaraciones.*		
26:1	*[...] información sobre mi tratamiento (punto positivo)*		
28:1	*[...] tener el historial completo de mi diagnóstico (punto positivo).*		
47:1	*Me pareció interesante el relato de todo mi historial desde el día de la cirugía.*		
29:2	*Me pareció un material de calidad, informativo y práctico.*		
36:2	*[...] cuidado y aplicabilidad en el día a día.*		
39:1	*[...] dejar claro que es posible tener una vida después del cáncer, sentirse mujer.*	Sentimientos positivos: conocimiento, seguridad, autoconfianza, reconocimiento de la iniciativa, de la necesidad de ser un derecho	Aceptabilidad: valioso
39:2	*[...] el plan me hizo bien.*		
39:4	*Continúen haciendo este trabajo. Después de recibir el plan hice un ensayo fotográfico.*		
58:1	*Muy válida la orientación para seguir la vida con confianza y alegría.*		
45:1	*[...] el contenido ayuda mucho.*		
47:4	*Felicitaciones por el excelente trabajo y por el cariño.*		
45:3	*Me pareció muy buena la idea, sugiero que continúe.*		
56:1	*[...] sobreviviente de cáncer es muy fuerte.*		
42:3	*Todos con cáncer deberían recibir el manual.*		
39:1	*[...] dejar claro que es posible tener una vida después del cáncer, sentirse mujer.*		
32:2	*[...] me gustó.*		
33:2	*[...] seguridad.*	Sentimiento positivo: seguridad	
18:2	*Muchas cosas por aprender.*	Facilita el aprendizaje	Aceptabilidad: educativo
23:1	*Me gustó la organización y los esclarecimientos.*		
53:2	*Me hizo aprender.*		
25:1	*[...] aclaración de la conducta y cuidados postratamiento.*		
54:1	*[...] mejora el conocimiento sobre su enfermedad.*		
36:1	*Lenguaje de fácil entendimiento.*	Contenido y lenguaje adecuados	Viabilidad: didáctico
22:1	*Organización del material y de la forma en que orienta.*		
38:1	*Lectura fácil, textos objetivos.*		
40:2	*[...] información clara.*		
46:1	*La disposición de los temas es de fácil uso.*		
21:2	*Lenguaje claro de los términos (punto positivo).*		
46:1	*La disposición de los temas es de fácil uso.*		
17:1	*[...] las aclaraciones son objetivas.*	Más allá de lo biológico	Aceptabilidad: holístico
41:1	*[...] no recuerdo todo lo que leí.*		
43:1	*Punto positivo: salud y bienestar.*		
20:1	*[...] cuidados con la mente.*		
41:2	*[...] excelente para quien está al inicio del tratamiento.*	Mejor aplicar al inicio del tratamiento	Aceptabilidad y viabilidad relativizadas: anticipar la entrega
41:3	*[...] superficial para quien está en una fase del tamoxifeno.*	Superficial en algunos aspectos	Aceptabilidad y viabilidad relativizadas: incompleto
41:4	*[...] pandemia y crisis de gripe y ese control de la salud también podría incluirse en el manual.*		
54:2	*Tener una cartilla adecuada para cada caso.*	Personalización/Individualización	Aceptabilidad y viabilidad relativizadas: individualizar
28:2	*No usaría la palabra sobreviviente.*	Incomodidad con el término “sobreviviente”	Aceptabilidad y viabilidad relativizadas: sobreviviente como denominación
56:1	*Sobreviviente de cáncer es muy fuerte.*		
48:1	*No leí el plan porque no me gusta leer y tampoco me gusta recordar mi enfermedad.*	Cantidad excesiva de contenidos	Aceptabilidad y viabilidad relativizadas: extenso

*TSSCP-P Br = Resumen del Tratamiento y Plan de Cuidados para la Sobreviviente de Cáncer de Mama

**Tabla 2 –:** Evaluación de la aceptabilidad y viabilidad del TSSCP Br*, desde la perspectiva de los profesionales (n^†^= 10). São Paulo, SP, Brasil, 2022

**Dimensión**	**Afirmativa**	**Evaluación**				
		**Totalmente en desacuerdo**	**En desacuerdo**	**Neutro**	**De acuerdo**	**Totalmente de acuerdo**
		**n** ^†^ **(%)**	**n** ^†^ **(%)**	**n** ^†^ **(%)**	**n** ^†^ **(%)**	**n** ^†^ **(%)**
Adecuación	Los objetivos de la intervención corresponden a los priorizados para las mujeres sobrevivientes de cáncer de mama.	0 (0,0)	0 (0,0)	0 (0,0)	3 (30,0)	7 (70,0)
	Las recomendaciones y orientaciones son aplicables a la población de sobrevivientes de cáncer de mama.	0 (0,0)	0 (0,0)	0 (0,0)	3 (30,0)	7 (70,0)
	La forma en que se está implementando la intervención es capaz de generar los objetivos deseados.	0 (0,0)	0 (0,0)	0 (0,0)	3 (30,0)	7 (70,0)
Conveniencia	La intervención es fácil de implementar en la práctica cotidiana.	0 (0,0)	1 (10,0)	0 (0,0)	4 (40,0)	5 (50,0)
	El equipo requiere entrenamiento específico para realizar la intervención.	0 (0,0)	0 (0,0)	0 (0,0)	4 (40,0)	6 (60,0)
Eficacia	La intervención es eficaz, tanto a corto como a largo plazo.	0 (0,0)	0 (0,0)	0 (0,0)	4 (40,0)	6 (60,0)
Riesgos	La intervención puede causar daño o malestar a la sobreviviente de cáncer de mama.	5 (50,0)	2 (20,0)	2 (20,0)	1 (10,0)	0 (0,0)
	La intervención causa riesgo para el profesional de la salud.	9 (90,0)	1 (10,0)	0 (0,0)	0 (0,0)	0 (0,0)
Adhesión	La sobreviviente de cáncer de mama hará uso del material para mejorar la vigilancia y los cuidados relacionados con la salud.	0 (0,0)	0 (0,0)	1 (10,0)	4 (40,0)	5 (50,0)
	El equipo de salud adoptará la intervención en la práctica cotidiana.	0 (0,0)	0 (0,0)	3 (30,0)	5 (50,0)	2 (20,0)
Disponibilidad, calidad y habilidad de los recursos humanos	La intervención requiere recursos humanos adicionales.	0 (0,0)	2 (20,0)	3 (30,0)	3 (30,0)	2 (20,0)
	Existen profesionales disponibles para aplicar la intervención.	0 (0,0)	1 (10,0)	1 (10,0)	7 (70,0)	1 (10,0)
	La intervención requiere recursos humanos especializados.	1 (10,0)	0 (0,0)	1 (10,0)	5 (50,0)	3 (30,0)
Entrenamiento	El equipo multiprofesional está capacitado para aplicar la intervención.	0 (0,0)	2 (20,0)	0 (0,0)	4 (40,0)	4 (40,0)
Recursos materiales, tecnológicos y físicos	El plan de cuidados en formato impreso es adecuado.	0 (0,0)	1 (10,0)	0 (0,0)	1 (10,0)	8 (80,0)
	Los recursos tecnológicos para el acceso a la información sobre la intervención son adecuados.	0 (0,0)	0 (0,0)	0 (0,0)	3 (30,0)	7 (70,0)
	El ambiente físico para la realización de la intervención es adecuado.	0 (0,0)	1 (10,0)	1 (10,0)	4 (40,0)	4 (40,0)
Fidelidad	El contenido en el plan de cuidados es de fácil comprensión.	0 (0,0)	0 (0,0)	0 (0,0)	4 (40,0)	6 (60,0)
	Las actividades relacionadas con el llenado del plan de cuidados son fáciles.	0 (0,0)	0 (0,0)	1 (10,0)	5 (50,0)	4 (40,0)
	Las actividades relacionadas con la aplicabilidad del plan son fáciles.	0 (0,0)	0 (0,0)	0 (0,0)	4 (40,0)	6 (60,0)
	El tiempo para ejecutar la intervención, considerando el llenado y la aplicación, es adecuado.	0 (0,0)	0 (0,0)	0 (0,0)	7 (70,0)	3 (30,0)
Alcance	Las actividades abarcan cuidados en las diversas fases de la supervivencia.	0 (0,0)	0 (0,0)	1 (10,0)	4 (40,0)	5 (50,0)

*TSSCP-P Br = Resumen del Tratamiento y Plan de Cuidados para la Sobreviviente de Cáncer de Mama; ^†^n = Número de participantes

De los diez participantes, siete (70%) escribieron comentarios sobre el TSSCP-P Br. Se empleó la técnica de análisis de contenido como en el grupo de respondientes de las mujeres sobrevivientes. Los contenidos de las citas correspondientes a los análisis inferenciales y sus respectivos códigos, referentes a la percepción de los profesionales sobre el TSSCP-P Br, están discriminados en la Figura 2.

**Figura 2 -:** Contenidos de citas correspondientes a los análisis inferenciales y sus respectivos códigos, referentes a la percepción de los profesionales sobre el TSSCP-P Br*. São Paulo, SP, Brasil, 2022

**ID**	**Contenido de cita**	**Análisis inferencial**	**Categorías**
4:5	*[...] información en la palma de la mano.*	Satisfacción profesional	Aceptabilidad: contribuye al ejercicio profesional
4:3	*[...] tamaño de bolso.*		
4:6	*[...] versión digital o física.*		
1:1	*Fue una experiencia increíble poder contribuir ademásaprender mucho con todos.*		
3:4	*[...] expresiones de gratitud y acogida que involucran a la familiaal propio paciente.*		
3:7	*Oportunidad de formar parte de este proceso.*		
4:1	*[...] placentero.*		
7:1	*Experiencia personal y profesional muy especial.*		
2:5	*[...] un “vínculo” entre paciente y profesional.*		
3:3	*[...] el plan incentiva el planteamiento de preguntas.*	Favorece la actividad laboral	
5:3	*Seguimiento del progreso del tratamiento.*		
5:2	*[...] facilitador en consultas futuras.*		
2:1	*[...] una gran herramienta no solo de información (...)*		
3:5	*[...] en las pacientes que abordé(...) hubo expresiones de gratitud y acogida, que involucraron a la familia y al paciente.*	Calidad en el cuidado	Viabilidad: cuidado centrado en el paciente y la familia
2:4	*[...] brinda acogida y cuidado a la paciente.*		
6:1	*[...] la mirada del profesional de la salud hacia la sobreviviente que ya ha pasado por el proceso más difícil de la enfermedad: descubrimiento, tratamiento y sufrimiento físico y social.*		
6:2	*[...] empático. Nosotros, profesionales, olvidamos que la vida de la sobreviviente cambia. Es necesario dar orientaciones para llevar la vida de otra manera.*		
5:1	*Fundamental importancia para la paciente, ya que trae los datos desde el inicio del tratamiento.*		
3:5	*[...] en las pacientes que abordé [...] hubo expresiones de gratitud y acogida, que involucraron a la familia y al paciente.*		Viabilidad: considera las implicaciones de la enfermedad oncológica en la sobreviviente
4:4	*[...] forma de aplicación.*	Con uso de tecnología digital	Aceptabilidad relativizada: forma de presentación
4:5	*Información en la palma de la mano.*	Presentación compacta	
4:3	*[...] tamaño de bolso.*		
4:6	*Versión digital o física.*	Con más de una presentación
4:2	*El Plan contiene toda la información, sin embargo,ser más compacto.*	Completo, con potencial para ser resumido	Viabilidad relativizada: exceso de contenidos
3:1	*[...] dos pacientes se sintieron incómodos conpalabra sobreviviente.*	La palabra “sobreviviente” puede generar incomodidad en la paciente	Viabilidad relativizada: “sobreviviente” como denominación

*TSSCP-P Br = Resumen del Tratamiento y Plan de Cuidados para la Sobreviviente de Cáncer de Mama

## Discusión

El estudio presentó datos que refuerzan la aceptación y viabilidad del TSSCP-P Br como recurso viable y calificador de la asistencia a mujeres sobrevivientes de cáncer de mama. La mayoría informó que el plan contenía información nueva, que la información contenida en él propició alguna toma de decisiones para planificar los cuidados de seguimiento y que las recomendaciones y orientaciones eran fáciles de ejecutar. En un estudio que evaluó el impacto del suministro de planes de cuidados para sobrevivientes, también se identificó que la mayoría de los participantes consideraron la información del plan como nueva^(9,20)^.

Los sobrevivientes de cáncer valoran intervenciones con contenido específico para sus necesidades, que sean fáciles de utilizar, accesibles y entregadas en el momento adecuado durante la trayectoria del cáncer^(21)^. Es importante destacar que las necesidades y preferencias varían de persona a persona en todas las fases de la trayectoria, incluido el período postratamiento, y que todos los sobrevivientes necesitan información integral y relevante para orientarlos en la gestión de la salud^(7,20)^.

Según lo constatado en este estudio, el TSSCP-P Br puede adoptarse como una guía de gran utilidad en todas sus partes constituyentes, tanto a corto como a largo plazo de la supervivencia, para dar seguimiento a la salud y el bienestar. Los datos corroboran estudios que indican que la mejora del conocimiento del paciente sobre su enfermedad y tratamiento genera un mayor compromiso con el proceso de gestión de la salud^(22-23)^. Esto también se alineó con los resultados satisfactorios en términos de viabilidad, con alta adhesión y retención, lo que demostró una mayor participación en el proceso de autogestión de la salud.

Para las enfermeras involucradas en la implementación, el TSSCP-P Br fue considerado viable, aunque se señalaron algunos factores limitantes, especialmente en relación con el número de profesionales y la especificidad técnica de estos en el área de oncología. La necesidad de recursos humanos complementarios puede justificarse por el tiempo requerido para la entrega del plan, que puede variar de 20 a 90 minutos según la literatura^(20)^. Además, la formación inadecuada de los profesionales para el cuidado de sobrevivientes de cáncer también es un factor limitante ampliamente discutido y que puede impactar negativamente en la práctica clínica^(20)^. Es importante destacar que la percepción de los profesionales al aplicar una intervención está influenciada por diversos factores, como valores y creencias personales, formación profesional, conocimiento teórico, experiencia práctica y el uso de directrices de buenas prácticas^(16)^.

Cuando los profesionales consideran inaceptable una intervención para los pacientes, pueden evitarla; por lo tanto, la percepción sobre la habilidad para ejecutarla, así como la practicidad en su aplicación, pueden aumentar la motivación para llevar a cabo la intervención, impactando en la fidelidad para la adopción de las acciones contenidas en el plan^(17)^.

La implementación de intervenciones exitosas proviene de la aceptación por parte de los usuarios. La aceptabilidad es un constructo multifacético que refleja hasta qué punto las personas que realizan o reciben una intervención de salud la consideran apropiada. El marco teórico de la aceptabilidad está compuesto por siete componentes: actitud afectiva, sobrecarga, aplicabilidad, ética, coherencia de la intervención, costos y autoeficacia^(12)^.

Las posibilidades analíticas de los testimonios evidenciaron que las respuestas discursivas fueron sumamente esclarecedoras, pues revelaron percepciones que no pudieron explorarse en las respuestas en escala de Likert, aumentando la validez de los hallazgos^(9,15)^.

En el análisis cualitativo de los testimonios sobre los atributos del TSSCP-P Br, desde la perspectiva de las mujeres sobrevivientes de cáncer de mama, se generaron categorías que expresaron diversas cualidades del instrumento. Esta población valora el acceso a información de autocuidado organizada en base al continuo del cáncer, de forma que apoye el autogestión^(24)^.

Desde el punto de vista de los profesionales que aplicaron la intervención, también se destacaron varios aspectos positivos, principalmente como una posibilidad de materialización del cuidado centrado en el paciente. En una investigación que tuvo como objetivo desarrollar y evaluar el TSSCP-S, los evaluadores (profesionales y mujeres sobrevivientes de cáncer de mama) observaron que su uso propició el cuidado centrado en el paciente^(25)^.

El cuidado centrado en la persona se encuentra entre los principales modelos que contribuyen a la excelencia de la asistencia^(26)^, defendido por instituciones reguladoras en salud y por aquellas que cuentan con programas de acreditación hospitalaria, como el *Sistema Brasileiro de Acreditação* (SBA-ONA), el de la *Joint Commission International* (JCI), basado en el modelo norteamericano; y el de la Acreditación Canadiense (*Accreditation Canada International* - ACI), recientemente renombrado como *Health Standards Organization* (HSO)^(27)^.

Cabe destacar que la institución sede del estudio está acreditada por *Accreditation Canada International* y esto significa que los resultados de esta investigación, al ser incorporados en la práctica cotidiana, impulsarán los logros a niveles aún superiores.

Además, para ambos grupos de respondientes se revelaron aspectos negativos del TSSCP-P Br, tales como el posible sufrimiento generado por información que anticipa hechos y riesgos, la generalidad y extensión, y el empleo del término “sobreviviente”. El término “sobreviviente de cáncer” es ampliamente utilizado por diferentes personas, instituciones de salud, organismos académicos y organizaciones políticas. Sin embargo, en muchos países, los pacientes interpretan estos términos de forma negativa, asociándolos al recuerdo del alto riesgo de muerte y a la disociación de la cura, lo que genera rechazo, ya que continúan lidiando con el miedo a la recurrencia^(28)^.

Comparando los hallazgos con la literatura, un metaanálisis que examinó la viabilidad de la implementación de planes de cuidados para sobrevivientes de cáncer, desde las perspectivas tanto de los sobrevivientes como de los profesionales de la salud, concluyó que los planes son aceptables y valorados por ambos^(20)^. En fin, evaluar la aceptabilidad y viabilidad puede ayudar a identificar facilitadores y barreras para la implementación de la intervención y comprender el alcance de los resultados esperados, como lo atestigua una vasta literatura sobre el tema^(16,29)^.

Entre las limitaciones, se destaca la recolección en una única sede y que la institución en cuestión se encuentra en la categoría de *cancer center*, con acreditación hospitalaria. Lamentablemente, esta no es la realidad de la mayoría de los centros de tratamiento públicos del cáncer en Brasil, que se encuentran en hospitales generales, no acreditados en Oncología, con un número limitado de profesionales especializados y estructuras físicas deficientes para una asistencia de excelencia.

Otra importante limitación es la caracterización sociodemográfica de las sobrevivientes de cáncer de mama, que presentan un perfil distinto al del escenario nacional, dificultando la generalización de los resultados para las sobrevivientes exclusivas del sistema público de salud brasileño. Un mayor tamaño muestral de profesionales también debe considerarse en estudios futuros para confirmar los hallazgos.

Desde la perspectiva de las contribuciones del presente estudio para el perfeccionamiento de la ciencia de la enfermería, cabe destacar la importancia de realizar investigaciones que evalúen la aceptabilidad y viabilidad de propuestas innovadoras. El estudio de aceptabilidad y viabilidad aportó, de hecho, un conjunto de datos que puede favorecer el refinamiento de la aplicación del TSSCP-P Br en el cuidado de mujeres sobrevivientes de cáncer de mama tras el término del tratamiento inicial.

## Conclusión

Los datos cuantitativos de este estudio indicaron que el TSSCP-P Br alcanzó niveles satisfactorios de aceptabilidad y viabilidad. Desde el punto de vista de las mujeres sobrevivientes de cáncer de mama, la viabilidad presentó valores elevados para retención y adhesión, así como para la aceptabilidad en todas las dimensiones. Desde la perspectiva de los profesionales, la aceptabilidad y viabilidad también mostraron valores elevados en casi todas las dimensiones evaluadas.

En el análisis cualitativo, las mujeres sobrevivientes de cáncer de mama elogiaron el TSSCP-P Br, evidenciando lo valioso, educativo y capaz de resumir información importante que resulta para favorecer la autogestión y ser didáctico. No obstante, también se señaló el riesgo de que resulte incompleto o extenso, además de manifestar incomodidad con el uso del término “sobreviviente”.

La evaluación de las enfermeras sobre el TSSCP-P Br también fue positiva, ya que favorece el ejercicio profesional cualificado, el cuidado centrado en el paciente y la familia, y es capaz de reunir la complejidad de la enfermedad oncológica en el contexto de la supervivencia. Sin embargo, las profesionales sugirieron la posibilidad de compactar contenidos, ponerlo a disposición en formato digital y advirtieron sobre el riesgo de que el término “sobreviviente” genere incomodidad en las pacientes.
